# Forget the Folk: Moral Responsibility Preservation Motives and Other Conditions for Compatibilism

**DOI:** 10.3389/fpsyg.2019.00215

**Published:** 2019-02-07

**Authors:** Cory J. Clark, Bo M. Winegard, Roy F. Baumeister

**Affiliations:** ^1^Department of Psychology, Durham University, Durham, United Kingdom; ^2^Department of Psychology, Marietta College, Marietta, OH, United States; ^3^Department of Psychology, Florida State University, Tallahassee, FL, United States; ^4^School of Psychology, The University of Queensland, St Lucia, QLD, Australia

**Keywords:** free will, determinism, compatibilism, motivated cognition, experimental philosophy, folk beliefs

## Abstract

For years, experimental philosophers have attempted to discern whether laypeople find free will compatible with a scientifically deterministic understanding of the universe, yet no consensus has emerged. The present work provides one potential explanation for these discrepant findings: People are strongly motivated to preserve free will and moral responsibility, and thus do not have stable, logically rigorous notions of free will. Seven studies support this hypothesis by demonstrating that a variety of logically irrelevant (but motivationally relevant) features influence compatibilist judgments. In Study 1, participants who were asked to consider the possibility that our universe is deterministic were more compatibilist than those not asked to consider this possibility, suggesting that determinism poses a threat to moral responsibility, which increases compatibilist responding (thus reducing the threat). In Study 2, participants who considered concrete instances of moral behavior found compatibilist free will more sufficient for moral responsibility than participants who were asked about moral responsibility more generally. In Study 3a, the order in which participants read free will and determinism descriptions influenced their compatibilist judgments–and only when the descriptions had moral significance: Participants were more likely to report that determinism was compatible with free will than that free will was compatible with determinism. In Study 3b, participants who read the free will description first (the more compatibilist group) were particularly likely to confess that their beliefs in free will and moral responsibility and their disbelief in determinism influenced their conclusion. In Study 4, participants reduced their compatibilist beliefs after reading a passage that argued that moral responsibility could be preserved even in the absence of free will. Participants also reported that immaterial souls were compatible with scientific determinism, most strongly among immaterial soul believers (Study 5), and evaluated information about the capacities of primates in a biased manner favoring the existence of human free will (Study 6). These results suggest that people do not have one intuition about whether free will is compatible with determinism. Instead, people report that free will is compatible with determinism when desiring to uphold moral responsibility. Recommendations for future work are discussed.

## Introduction

A recent Marist Poll (2011) found that 73% of American citizens below the age of 45 believe in soul mates. Now imagine this piqued the interest of philosophers and they began to debate soul mates. Careful, rigorous analysis by philosophers might yield differences of opinion as to whether soul mates entail immaterial souls or are compatible with a purely materialistic concept of human mating. Some might then collect nuanced data about what laypersons believe about soul mates. But perhaps layperson beliefs are not based on scrutiny of metaphysical assumptions and instead stem from romantic notions, Hollywood movies, attempts to rationalize romantic breakups, or mere wishful thinking.

The present research was driven by the hypothesis that layperson responses to experimental philosophy questions about free will and compatibilism may likewise yield answers reflecting assorted motives and romanticized notions. Efforts to establish whether the folk are naïve compatibilists or incompatibilists may misfire, in part because people fail to understand the theoretical issues and base their answers on factors other than what philosophers seek to illuminate. Specifically, we will argue that desires to uphold free will and moral responsibility influence judgments about compatibilism.

### The Scholars

Among professional academic philosophers, there is a divide over whether free will and moral responsibility are compatible with a deterministic universe, that is, a universe in which the laws of nature and a complete description of the universe at any point in time logically entail the state of the universe at any other point in time (definition from Haggard et al., 2015). Those who believe free will and moral responsibility are compatible with a deterministic universe (compatibilists) typically define free will as the ability to perform actions on the basis of rational deliberation in the absence of coercion, whereas those who believe that determinism precludes free will (incompatibilists) typically define free will as entailing the genuine possibility of acting in different possible ways despite precisely the same causal history (also called libertarian free will; Haggard et al., 2015)^1^.

Incompatibilists argue that without alternative possibilities for action, people cannot be fully responsible for their actions (e.g., van Inwagen, 1983; Pereboom, 2006). Whereas compatibilists argue that their more lenient version of free will is sufficient for moral responsibility (e.g., Frankfurt, 1969; Wolf, 1990; Dennett, 2004). Virtually everyone agrees that humans have the ability to perform actions on the basis of rational deliberation. The disagreement is whether this kind of freedom deserves the name “free will,” a term that imbues human behavior with moral significance (though see Fischer (2007) for an alternate view of moral responsibility). Note that both incompatibilists and compatibilists agree that free will (whatever it is) is a necessary (but probably not sufficient) condition for moral responsibility.

### The Folk

In recent years, experimental philosophers have begun testing the intuitions of common people (often called “the folk”) regarding the necessary features of moral responsibility. To the surprise of many, this work has demonstrated contradictory results regarding lay intuitions about free will. For example, Nahmias et al. (2005) found that the majority of participants judged that specific actors acted of their own free will and were morally responsible in deterministic scenarios. Nichols and Knobe (2007) later suggested that compatibilist responses may represent affective reactions to specific stories about immoral behaviors and that in a more abstract, theoretical context, people are largely incompatibilists.

Continuing the debate, Murray and Nahmias (2014) argued that incompatibilist responses are due to a mistaken view of determinism that assumes that mental states are bypassed in the chain of events leading to human behavior, a misunderstanding termed ‘bypassing.’ In a meta-analysis of 30 studies, Feltz and Cova (2014) concluded that affective reactions may have little effect on compatibilist responding. Others have outlined myriad methodological challenges such as long and complicated stimuli and misunderstandings about the inevitability of causally determined outcomes (e.g., Turri, 2017a). The original 2005 paper by Nahmias and colleagues has now been cited 336 times (as of this sentence being written). That high number is partly a tribute to the importance of their work but also reflects the continuing, unresolved debate.

### The Problem

We propose one explanation for the discrepant findings regarding folk compatibilism: People are motivated to uphold their beliefs in free will and moral responsibility. Most laypersons believe in free will (e.g., Sarkissian et al., 2010) and disbelieve determinism (Nichols, 2004; Nichols and Knobe, 2007; Turri, 2017b), and people tend to evaluate information in a biased manner that favors their desired beliefs (e.g., Lord et al., 1979; Kunda, 1990). Thus, people are likely to report that free will is possible regardless of experimentally imposed constraints (Feltz and Millan, 2013), particularly when desiring to uphold moral responsibility (Clark et al., 2014, 2017).

#### Motivated Beliefs

Judgments with moral significance (an unavoidable feature of compatibilism judgments) are particularly fertile ground for motivated cognition (e.g., Haidt, 2001; Ditto et al., 2009; Clark et al., 2015). Though motivated cognition has a variety of meanings (see Kunda, 1990), here, we mean reasoning processes that favor preferred conclusions such that conscious beliefs and attitudes are shaped by desires. In other words, what people want to believe is true affects what they actually believe is true. Even when it comes to logic-based judgments, people seem incapable of fully escaping the influence of their own prior beliefs and desired truths: People are more likely to rate classically structured logical syllogisms as sound when the conclusions are consistent with their moral beliefs than when they oppose their beliefs, regardless of their actual soundness (Gampa et al., 2016). In compatibilism research, people likely struggle with the hypothetical analysis that requires accepting the apparently false premise of determinism.

The vast majority of people believe strongly in free will (Nadelhoffer et al., 2014) and that our universe is indeterministic (Nichols and Knobe, 2007). In fact, children as young as 4 years old demonstrate a belief in the ability to do otherwise (Nichols, 2004). It is problematic, then, that most (if not all) compatibilism studies (e.g., Nahmias et al., 2005, 2006, 2007; Nichols and Knobe, 2007; Feltz and Cokely, 2008; Roskies and Nichols, 2008) first present participants with a description of determinism (which they would be motivated to reject) and then ask whether free will and moral responsibility are compatible with that described universe (which they would be motivated to accept). People cannot be expected to logically and dispassionately evaluate arguments with apparently false premises, especially when those premises pose potential threats to core beliefs such as free will and moral responsibility (much like you wouldn’t expect a devout Christian to make rational judgments about creationism).

#### Motivated Beliefs in Free Will and Moral Responsibility

In addition to this complication, people tend to attribute more responsibility to individuals when motivated to hold them morally responsible. For example, past work has shown that people attributed more causal control to a speeding driver in a car accident when his reason for speeding was nefarious than morally admirable (Alicke, 1992), and more intention and personal causality to performers of actions with harmful consequences than performers of identical actions with helpful consequences (e.g., Knobe, 2003, 2006; Leslie et al., 2006; Knobe and Fraser, 2008).

Most problematic for compatibilism research, people attribute more free will to performers of morally bad actions than morally good actions and morally neutral actions (Clark et al., 2018; Everett et al., 2018; Feldman et al., 2016), and pondering morally bad actions leads people to increase their belief in the free will of all humankind (Clark et al., 2014). Thus, not only are people motivated to maintain the possibility of free will because they generally believe in free will, but they are particularly likely to assert free will when their judgment has moral significance, as compatibilism judgments do.

There is already quite a bit of evidence that motivations to maintain human moral responsibility influence compatibilist responding, though such results are not always interpreted this way. For example, Nichols and Knobe (2007) found that participants who were asked to evaluate an immoral behavior were far more compatibilist than participants asked to evaluate moral responsibility in an abstract manner. This spurred a debate about whether affect influences compatibilist judgments, with some studies finding no or small effects of affect on compatibilist judgments (e.g., Feltz et al., 2009; Feltz and Millan, 2013; Feltz and Cova, 2014). However, in these subsequent studies, both the high and low affect conditions involved morally bad behaviors that simply varied in severity. Therefore, both conditions likely activated motivations to maintain moral responsibility. This raises an important distinction between prior work and the present work. We do not contend that generalized affect necessarily influences free will judgments (though it might). Rather, we contend that compatibilist judgments may often reflect bias toward preserving free will, particularly so as to preserve moral responsibility (though there are likely many reasons and motives for free will beliefs).

Consistent with this suggestion, Roskies and Nichols (2008) found that people were far more likely to report that free choice and moral responsibility were possible in a deterministic universe if that deterministic universe was their own (i.e., the universe inhabited by humans) than if it were an alternate universe. Again, these results suggest that people are primarily concerned with preserving the ability to hold others (fellow humans) morally responsible. As suggested by Roskies and Nichols, “the intuition that we are in fact morally responsible is a non-negotiable intuition.” We agree, and this is precisely why the folk are incapable of evaluating deterministic contexts for free action in a rational, philosophically sophisticated manner.

Motives to hold people morally responsible may even influence how people interpret determinism. In Nahmias et al. (2005), all participants were given identical descriptions of a supercomputer that can predict the entire state of the universe at any point in time with 100% accuracy. For some participants, the supercomputer then accurately predicted an immoral behavior (a bank robbery), whereas others read that the supercomputer accurately predicted a neutral (jogging) or positive behavior (saving a child). Only in the case of the morally bad behavior did the majority of participants insist that the actor had the possibility of not performing the behavior. This may mean that people reject deterministic descriptions that assert the inability to do otherwise when motivated to hold others morally responsible. Even if experimental philosophers can get participants to comprehend determinism, participants may be unwilling to accept and apply the premise, particularly when evaluating moral responsibility for misdeeds.

Some have suggested that motivated reasoning can even be found among the most disciplined and sophisticated thinkers, namely philosophers. Schwitzgebel and Cushman (2012) found that the moral judgments of professional academic philosophers were equally if not more unstable than those of non-philosophers. This is in line with the expertise paradox in the motivated reasoning literature (e.g., Taber and Lodge, 2006; Kahan et al., 2012; Liu, 2013; Liu and Ditto, 2013): perceiving oneself as more knowledgeable and gaining more knowledge on a particular issue can actually increase biased responding, because expertise in a particular area can lead to more skillful *post hoc* rationalization of intuitive moral judgments (e.g., Haidt, 2001). In fact, one analysis suggests that compatibilism emerged among philosophers because it is motivationally attractive, specifically, to philosophers who have accepted determinism. As noted by Nichols (2007), among the twenty major philosophers of the early modern period, not one indeterminist was compatibilist. This suggests that when people acknowledge the possible reality of determinism, they may construct compatibilism as a means of avoiding the potential threat to moral responsibility.

#### Comprehension and Ambiguity

One final reason to expect compatibilist judgments to be particularly vulnerable to motivated reasoning is their complicated and abstract nature. Even professional philosophers struggle to agree on definitions of the central concepts (i.e., free will, the ability to do otherwise) and the proper words to use (or avoid) in thought experiments. Participants in compatibilism research have great difficulty comprehending exactly what determinism entails, and ambiguity increases biased responding (e.g., Dunning et al., 1989). Many of these studies do not measure or report comprehension failure rates, but of those that do, 10–60% of participants fail comprehension checks about determinism (e.g., Nichols and Knobe, 2007; Murray and Nahmias, 2014). Given that some participants likely answer correctly by guessing, it can be assumed that even more fail to fully understand the determinism scenarios in these studies, despite the effort and care experimental philosophers put into writing these descriptions.

The term free will is likely not a rigorously defined philosophical term. There are probably many folk theories that vary both between (Feltz and Cokely, 2009; Schulz et al., 2011) and within individuals (Deery et al., 2014), and it is probable that many of them are inconsistent with each other. Because of the abstract and complex nature of free will, the folk are likely incapable of furnishing meaningful conclusions about the concept. It is quite possible that a rudimentary notion of “free will” is a natural output of the human brain (see McCauley, 2011 for a discussion of “natural”), but that this representation is not philosophically coherent. This intuition is then complicated by more abstract teachings and discussions of free will as one would encounter among friends and in the news (say, about free will and the latest results of neuroscience). The resulting belief is likely an incoherent series of intuitions about free will that change from setting to setting (and, in particular, between everyday conversations and more elevated philosophical discourse).

### Integrative Summary and Present Research

In the present paper, we contend that responses to abstract dilemmas posed in experimental philosophy thought experiments may be guided less by careful reasoning (a point that many would concede) than by desires to uphold free will and moral responsibility. Though likely many feelings, attitudes, and desires influence free will beliefs, we suggest that motives to uphold moral responsibility are one reason people assert their belief in free will.

Overall, we find the current state of the debate best characterized by Deery et al. (2014): Most people are probably both compatibilists and incompatibilists, and though these positions appear contradictory, they are likely not psychologically contradictory. Philosophers have devoted a great deal of careful thought and debate to various specific definitions of free will, whereas the folk likely have not given it much thought at all and are reasonably satisfied with the conclusion that it exists, regardless of what it is. Indeed, some philosophers have suggested that people have *free-will-no-matter-what* intuitions (Feltz and Millan, 2013) and non-negotiable beliefs in the human capacities to make choices and control their actions (Nahmias and Thompson, 2014). This may mean that people will assert that free will and moral responsibility exist regardless of deterministic or fatalistic constraints.

To summarize, people believe strongly in human free will and that the universe is not deterministic. People are also motivated to maintain moral responsibility, particularly when considering concrete instances of moral behavior. Crucially for the present analysis, these beliefs and motives may irrationally constrain related judgments. Therefore, it is unclear whether people ascribe free will to actors in deterministic scenarios because of rational, compatibilist considerations regarding the necessary features of free will and moral responsibility–or because they reject the premise of a deterministic universe and are motivated to maintain the possibilities of free will and moral responsibility. In many cases, they may not even be able to comprehend the counterfactual deterministic world they are asked to consider. Unlike philosophers, everyday people do not give much thought to these complicated philosophical issues, and their intuitions are therefore of limited philosophical significance (e.g., Mele, 2003, 2008). To be sure, they are not completely useless, but they probably have more psychological than philosophical significance.

Across seven studies, we demonstrate that compatibilist judgments are influenced by a variety of factors that are irrelevant to the logical compatibility of determinism and moral responsibility. Instead, such judgments often stem from motives to preserve human free will and moral responsibility. Overall, our results highlight the need for extreme caution in interpreting the responses of ordinary people to philosophical questions regarding free will, moral responsibility, and determinism, because their responses are characterized by misunderstandings and bias. We conclude with implications and recommendations for experimental philosophy.

## Study 1

Study 1 tested whether having people consider the possibility that they live in a deterministic universe (rather than an indeterministic one) would increase compatibilist responses. In principle, compatibilist notions ought to be unaffected by considerations of whether our universe happens to be deterministic. Compatibilist notions ought to be based purely on considerations regarding the features of physical causality and/or moral responsibility. If contemplating the possibility that our own universe is deterministic increases compatibilist responding, this would suggest that the possible truth of determinism poses a threat to moral responsibility, which participants then combat by being more compatibilist. Ordinary folk may not be alone in this. Nichols (2007) proposed that compatibilism became popular among determinist philosophers for much the same reason, namely the wish to preserve moral responsibility.

First, all participants read a paragraph stating that humans have free will and can be held morally responsible. Participants then read either an argument^2^ stating that the universe is deterministic or that it is indeterministic^3^. Then they were asked whether the second argument (either supporting determinism or indeterminism) could possibly be true if people have free will and can be held morally responsible as stated in the first argument. Last, participants in both conditions were asked whether determinism is possible if humans have free will and can be held morally responsible (i.e., whether compatibilism is possible). This second question was identical across the two conditions. The response to this question should be unrelated to which argument they read, so any differences between conditions might indicate something about the motivations arising from the possibility of determinism.

We first predicted that participants in the indeterminism condition would be more likely to state that their argument was possible if people have free will and can be held morally responsible than participants in the determinism condition. This, of course, would be unsurprising (and fairly uninteresting), as most philosophers (though not all) would concur that people can be morally responsible in an indeterminist universe^4^. However, we also predicted that participants in the determinism condition would be more compatibilist than participants in the indeterminism condition. If the possibility of determinism poses a threat to moral responsibility, people may be more likely to reconcile free will and determinism when asked to consider the realistic possibility that humans live in a deterministic universe. This would suggest that compatibilist responding is motivated, in part, by desires to uphold moral responsibility.

### Methods

In an online study, 106 undergraduates at a southwestern United States university (84 female, *M*_age_ = 20.55) first read a passage stating that humans have free will, “*…Humans are highly intelligent, complex beings with the capacity for free will… They sometimes deserve to be praised for good things they freely decide to do and deserve to be blamed for bad things they freely decide to do.*^5^”

Participants were then randomly assigned to read one of two passages, which argued either that the universe is deterministic, “…*All human thoughts and their corresponding behaviors are inevitable consequences of prior events. If past events had happened differently, then a person would have had different thoughts and desires, which would have led to different decisions”* or indeterministic, “…*Human thoughts and their corresponding behaviors are not always inevitable consequences of prior events. Even if past events had happened the exact same way, a person could have different thoughts and desires, which could lead to different decisions.*” Descriptions were written by the authors, though aspects are similar to Nichols and Knobe (2007).

Participants were then asked two comprehension questions: “According to the argument, do humans’ thoughts influence their behavior?,” the correct answer being “yes” in both conditions (henceforth referred to as bypass comprehension), and “According to the argument, if specific events occurred, would a human make a particular decision with 100% certainty?,” the correct answer being “yes” in the deterministic condition and “no” in the indeterministic condition (henceforth referred to as otherwise comprehension). Participants were also given “I don’t know” options.

Participants then responded to two questions that served as our main dependent variables. The first question asked whether the argument they read was compatible with free will: “If humans have free will and can be morally responsible as specified above, is it possible for the argument to be true?” with response options “yes,” “no,” and “I don’t know.” Thus, participants in the deterministic condition were asked whether determinism was compatible with free will and participants in the indeterminism condition were asked whether indeterminism was compatible with free will.

The second question (posed to all participants) simply asked whether determinism was compatible with free will: “If humans have free will and can be morally responsible as specified above, is it possible for all human behavior to be inevitable consequences of prior causes?” with response options “yes,” “no,” and “I don’t know.” Participants then completed the free will, scientific determinism, and fatalistic determinism subscales of the Free Will and Determinism Scale (FAD+; Paulhus and Carey, 2011), which measure beliefs in free will, scientific determinism, and fatalistic determinism. Last, participants responded to an attention check and variety of demographic questions.

### Results

#### Comprehension Checks

The majority of participants passed the bypass comprehension check in both the determinism condition (77.8%) and the indeterminism condition (82.7%). Participants had a more difficult time with otherwise comprehension. Whereas 80.8% of participants in the indeterminism condition correctly understood that even given past events, humans might not make a particular decision, only 40.7% of participants in the determinism condition correctly understood that if specific events occurred, humans would make a particular decision. Comprehension problems are a recurring issue in this and similar work. In the present work, it was not always feasible or possible to test for moderation by comprehension, nor was it always feasible or possible to report results separately for participants who passed comprehension checks (due to small cell sizes). Rather than report each set of results multiple times with various subsets of participants, all main results with comprehenders only are reported in Supplementary Material. No exclusions were made for any results reported in the main text.

#### Argument Compatibility

**FIGURE 1:**
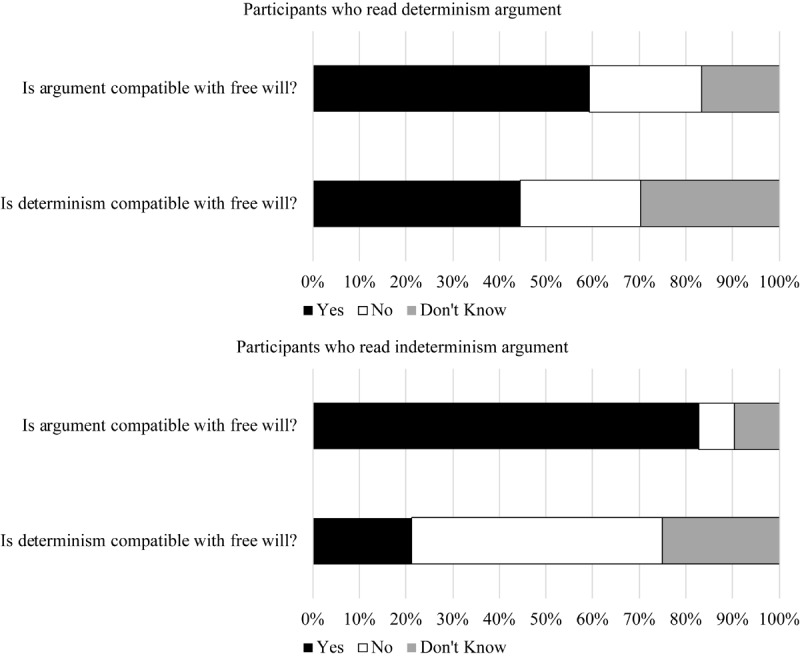
Argument compatibility and determinism compatibility responses within each argument condition in Study 1.

For Studies 1–3b, data were analyzed with multinomial logistic regression, a statistical test that allows for nominal dependent variables with more than two response options (e.g., “yes,” “no,” and “I don’t know”), and compares the relative rate of one response to another between levels of the independent variable. Participants in the indeterminism condition were significantly more likely to report that their argument was compatible with free will than that their argument was incompatible relative to those in the determinism condition, with a medium to large effect size (see Figure 1 and Table 1)^6^.

**Table 1:** Study 1 argument compatibility and determinism compatibility responses by condition.

	*Determinism condition*	*Indeterminism condition*	*OR*	*b*	*SE*	*Wald*	*p*	*95% CI*
**Argument compatibility**								
Yes	59.3%	82.7%						
No	24.1%	7.7%	4.37	1.47	0.62	5.70	0.017	1.30, 14.65
Don’t Know	16.7%	9.6%	2.42	0.88	0.61	2.13	0.144	0.74, 7.91
**Determinism compatibility**								
Yes	44.4%	21.2%						
No	25.9%	53.8%	0.23	−1.47	0.49	9.06	0.003	0.09, 0.60
Don’t know	29.6%	25.0%	0.56	−0.57	0.52	1.21	0.272	0.20, 1.57

*‘Yes’ response is reference category.*

#### Determinism Compatibility

Participants in the determinism condition were also significantly more likely to report that determinism was compatible with free will than that determinism was incompatible with free will relative to those who read the indeterminism argument, with a medium to large effect (see Figure 1 and Table 1). For participants in the indeterminism condition, the pattern was reversed with more participants saying determinism was incompatible with free will than that it was compatible.

### Discussion

Although participants were generally more likely to report that indeterminism was compatible with free will than that determinism was compatible with free will, these differences were fairly small, and the majority of participants (even those in the determinism condition) reported that their argument was compatible with free will. These results appear to suggest that people are intuitive compatibilists–though they also fit the view that people are mainly guided by the motivation to preserve belief in free will and moral responsibility. Consistent with the latter interpretation, our second dependent variable suggests that what appeared to be compatibilism may actually be motivated responding driven by desires to maintain belief in moral responsibility.

There were large differences in compatibilist attitudes between the two argument conditions on the more general question about whether determinism is compatible with free will. Participants who were previously asked to consider the possibility that they live in a deterministic universe maintained relatively high levels of agreement with compatibilism when asked about compatibilism more generally. In contrast, participants who had not previously considered the possible truth of determinism reported very low levels of agreement with compatibilism on the more general question.

These results support our hypothesis that compatibilist attitudes may often reflect desires to maintain the possibility of moral responsibility (including the associated assumption of free will) and may suggest that the mere consideration of the possibility of determinism leads people to change the rules about the necessary features of moral responsibility. When there was no threat of determinism (and hence no potential threat to moral responsibility), participants were much less likely to reconcile determinism with the capacity for moral responsibility.

It is also worth noting the high frequency of “I don’t know” responses and failures to comprehend determinism. We propose three possible explanations. (1) We did a poor job explaining determinism and/or asking the comprehension question. (2) Perhaps failure to comprehend the true meaning of determinism reflects rejection of its truth. (3) These are abstract concepts and participants simply do not understand them. This is a recurring issue with research in this area, and calls into question the value of conducting this kind of research in the first place. To address these various possibilities, subsequent studies used different paradigms involving different descriptions of determinism and question phrasing to capture a broader range of possible interpretations of these concepts.

A potential alternate explanation is that participants in the determinism condition were simply persuaded by the determinism argument and thereby effectively convinced that compatibilism was true. If so, participants in the determinism condition might not have been reporting more compatibilist attitudes due to motives to uphold moral responsibility, but rather because they were convinced that determinism is compatible with free will and moral responsibility. To address this, Study 2 manipulated motives to maintain moral responsibility.

## Study 2

Study 2 directly manipulated motives to hold others morally responsible in order to test whether such motives influence compatibilist responding. Past research has shown that motives to punish (Clark et al., 2014) and praise (Clark et al., 2018) underlie free will beliefs. Hence people should be more likely to agree that compatibilist free will is sufficient for moral responsibility when considering concrete morally virtuous and harmful actions than when contemplating abstractly (consistent with Nichols and Knobe, 2007). This would fit the overarching theme that people are primarily concerned with maintaining moral responsibility, and they adjust their beliefs and assumptions about free will on that basis.

In Study 2, participants read an argument that people have either libertarian free will or compatibilist free will. They were instructed to assume the argument is correct, and then were asked whether it makes sense to either hold people morally responsible in general (morally neutral condition), to morally blame someone (morally bad condition), or to morally praise someone (morally good condition). We predicted that participants would be more likely to report that libertarian free will is sufficient for moral responsibility than compatibilist free will in general. Furthermore, we predicted that there would be little to no differences in moral responsibility ratings between the three moral behavior conditions in the libertarian free will condition, as moral responsibility is already satisfied in this condition. However, in the compatibilist free will condition, participants in the morally praiseworthy and blameworthy conditions would be more likely to report that compatibilist free will is sufficient for moral responsibility than those in the neutral condition.

### Methods

In an online study, 370 undergraduates at a southwestern United States university (282 female, *M*_age_ = 19.97) were randomly assigned to read either a libertarian description of free will, “**…** *scientists have come to learn many influences on human behavior, including genes, brain chemistry, and social environment… However… these uncontrollable factors cannot entirely predict human behavior with 100% certainty… human behaviors are not inevitable consequences of prior causes and that humans have the capacity to choose among alternate courses of action…”* or a compatibilist description of free will, “*…scientists have come to learn many influences on human behavior, including genes, brain chemistry, and social environment… these uncontrollable factors entirely predict human behavior with 100% certainty… all human behaviors are inevitable consequences of prior causes… humans do have the capacity to act in accordance with their own thoughts and desires… thoughts and actions are links in a chain of causes.”* Descriptions were written by the authors, though aspects are similar to Murray and Nahmias (2014).

Participants were then asked a comprehension question, which contained a moral relevance manipulation. In the neutral condition, they were asked, “If these scientists are correct, is it possible for a person to behave differently than that person ultimately does?” In the morally bad condition, they were asked, “If these scientists are correct, is it possible that a man who murdered his wife in order to have an affair with his secretary could have not done so, that is, could he have not murdered his wife?” In the morally good condition, they were asked, “If these scientists are correct, is it possible that a man who donated 90% of his annual salary to a pediatric cancer center could have not done so, that is, could he have not donated 90% of his annual salary to the pediatric cancer center?” Across all three conditions, the correct answer was “yes” in the libertarian condition and “no” in the compatibilist condition (though note some compatibilist philosophers would disagree with this; we dealt with this by varying the phrasing of this comprehension check across studies).

Next, participants were asked the primary dependent measure. Participants in the neutral condition were asked, “If these scientists are correct, does it make sense to hold people morally responsible for their behavior?” Participants in the morally bad condition were asked, “If these scientists are correct, does it make sense to morally blame a man who murdered his wife in order to have an affair with his secretary?” Participants in the morally good condition were asked, “If these scientists are correct, does it make sense to morally praise a man who donated 90% of his annual salary to a pediatric cancer center?” Participants were then asked to explain their answer in an open response. Last, participants completed the same subscales of the FAD+ (Paulhus and Carey, 2011) as in Study 1, and responded to an attention check and variety of demographic questions.

### Results

#### Comprehension Checks

In the libertarian free will condition, 82.1% of participants correctly understood that the actor could have done otherwise, whereas, in the compatibilist free will condition, only 35.5% of participants correctly understood that the actor could not have done otherwise, once again demonstrating an incomprehension or rejection of the notion of inability to do otherwise.

#### Moral Responsibility

We conducted multinomial logistic regressions with the free will condition, dummy coded moral relevance conditions (reference category: neutral), and the interactions predicting moral responsibility responses. The interactions between the free will condition and the moral relevance dummy variables were not significant, *p*s > 0.280. Rather than the moral relevance manipulation having a larger effect in the compatibilist free will condition than the libertarian free will condition (as we had predicted), the moral relevance manipulation had roughly equivalent influence in both free will conditions. Because this part of our prediction appeared to be incorrect, the interactions were dropped from the model in order to interpret the main effects.

**Table 2:** Moral responsibility responses by free will and moral conditions in Study 2.

Free will condition	Moral condition	Yes	No	Don’t know
Compatibilist	Good	54.7%	39.1%	6.3%
Free will	Bad	41.7%	50.0%	8.3%
	Neutral	33.9%	46.8%	19.4%
Libertarian	Good	78.5%	9.2%	12.3%
Free will	Bad	86.4%	8.5%	5.1%
	Neutral	71.2%	13.6%	15.3%

**Table 3:** Moral responsibility compatibility results in Study 2.

	Comparison	*OR*	*b*	*SE*	*Wald*	*p*	*95% CI*
Free will condition	Yes vs. No	7.95	2.07	0.29	50.78	<0.001	4.49, 14.06
	Yes vs. DK	1.91	0.65	0.35	3.46	0.063	0.97, 3.77
Good dummy	Yes vs. No	1.73	0.55	0.32	2.88	0.090	0.92, 3.24
	Yes vs. DK	2.44	0.89	0.40	4.94	0.026	1.11, 5.34
Bad dummy	Yes vs. No	1.32	0.27	0.32	0.73	0.392	0.70, 2.46
	Yes vs. DK	3.20	1.16	0.45	6.64	0.010	1.32, 7.75

*‘DK’ stands for ‘Don’t Know’ response.*

**FIGURE 2:**
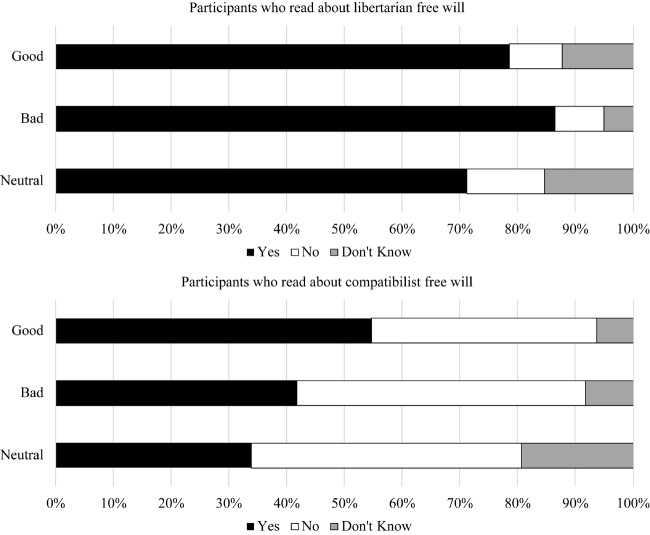
Responses to whether people can be held morally responsible given each free will type by each moral condition in Study 2.

As can be seen in Tables 2, 3 and Figure 2, participants were significantly more likely to report that libertarian free will was sufficient for moral responsibility than that it was insufficient for moral responsibility relative to participants in the compatibilist free will condition, with a large effect. Indeed, participants were generally more likely to report that compatibilist free will was insufficient for moral responsibility than that it was sufficient.

Participants in the morally good conditions were more likely to report that their version of free will was sufficient for moral responsibility than that it was insufficient for moral responsibility relative to participants not in the morally good condition with a small to medium effect. The strongest effect in the morally bad condition was a reduction in uncertainty such that participants in the morally bad condition were significantly more likely to say that their version of free will was sufficient for moral responsibility than that they did not know whether it was sufficient relative to those not in the morally bad condition with a small to medium effect.

### Discussion

Participants were far more likely to agree that libertarian free will was sufficient for moral responsibility than that compatibilist free will was sufficient. Furthermore, participants were more likely to report that their version of free will was sufficient for moral responsibility (than that it was not sufficient or that they did not know whether it was sufficient) in the conditions with moral relevance. These results, in combination with the results of Study 1, suggest that motives to maintain moral responsibility may be a motivating factor behind compatibilist responses.

Contrary to predictions, the moral relevance condition had roughly equal influence in the libertarian and compatibilist free will conditions. We expected a ceiling effect in the libertarian free will condition such that nearly all participants would agree that libertarian free will was sufficient for moral responsibility in all conditions. Though the majority of participants did agree that libertarian free will was sufficient for moral responsibility in the neutral condition (71.2%), still more agreed when considering concrete instances of morally good (78.5%), and morally bad (86.4%) behavior. Though this result was not predicted, it is still consistent with our general hypothesis that motives to preserve moral responsibility influence judgments about the very features required for moral responsibility.

## Study 3A

Study 3a investigated the troublesome possibility that judgments about the compatibility of two arguments would depend on which one they saw first. All participants were given identical free will and determinism arguments, but the order of presentation of the arguments was manipulated. Some participants were asked to assume the free will argument was true and then evaluate the compatibility of the determinism argument; others were asked to assume the determinism argument was true and then evaluate the compatibility of the free will argument. Of course, compatibility between two assumptions should be bidirectionally symmetrical. That is, if people are perfectly rational, there would be no difference based on the sequence of reading them. But if the guiding factor is that people are motivated to maintain moral responsibility, participants who read the free will argument first should be satisfied by the assumption that people have free will and hence willing to judge determinism on its own merits– whereas if they are first told to assume determinism, they would want to insist on the existence of free will by finding the two compatible. Hence the motivated reasoning perspective would predict an order effect, such that people insist on compatibilism when it is necessary to salvage free will – but would not care about salvaging determinism once free will is assumed. This was our main prediction.

As in Study 2, Study 3a also manipulated moral relevance. In both arguments, the final sentence described the implications for either a morally neutral behavior (a man having pasta for dinner) or a morally bad behavior (a man robbing a convenience store). Participants then reported whether it is possible for the second argument to be true *if the first argument is true*. We predicted the order effect would be stronger when contemplating the robbery than the eating of noodles. That is, people should be most motivated to maintain belief in free will–even if that means embracing compatibilism–when desiring to hold a bad guy accountable.

### Methods

In an online study, 356 undergraduates at a northeastern United States university (182 female, *M*_age_ = 19.06) were told they were going to evaluate the logical compatibility of two arguments. They were asked to read one argument, assume it was true (regardless of whether they personally agreed it was true), and then evaluate whether a second argument could then also be true, or whether the truth of the first argument meant the second argument was necessarily false. One argument supported the truth of determinism, “**…** *scientists have come to learn the many causes of human behavior, including genes, brain chemistry, and social environment, and have learned that these factors predict human behavior entirely. All human thoughts and their corresponding behaviors are inevitable consequences of prior events. People’s mental states (their beliefs, desires, and decisions)… are merely part of the causal chains that lead to their actions…”* whereas the other argued that people have free will and can be held morally responsible, “**…** *While many external and internal factors interact to influence human behavior, humans are capable of free action. Because humans have free will, they deserve to be held responsible for their behavior. When a person behaves in a particular way, they are responsible for having behaved that way.”* Descriptions were written by the authors, though aspects are similar to Murray and Nahmias (2014). The order of presentation was randomly assigned.

We also manipulated whether the argument had implications for a morally neutral behavior (a man having pasta for dinner) or a morally relevant action (a man robbing a convenience store). In each argument, the final sentence of the paragraph described the implications of that argument for either the morally neutral behavior (e.g., after the determinism argument, “*So, for example, if a man decides to have pasta for dinner and subsequently has pasta for dinner, given his genes, brain chemistry, and his social history, there is no way he could have not had pasta for dinner*”) or the morally relevant behavior (e.g., after the free will argument, “*So, for example, if a man robs a convenience store, he exercised his free will in robbing a convenience store and ought to be held morally responsible for that action*”).

Participants were then asked whether the second argument was compatible with the first. They were told that we were not interested in whether they personally believed either argument was true, but simply to evaluate whether they thought the second argument was logically compatible or incompatible with the first argument. The response options were a compatibilist response “If the first argument is true (that all human thoughts and actions are predetermined/that humans have free will and are morally responsible for their behavior), the second argument (that humans have free will and are morally responsible for their behavior/that all human thoughts and actions are predetermined) CAN also be true,” an incompatibilist response (CANNOT replaced the word CAN), or “I don’t know.” Participants were then asked how certain they were that their answer was correct on a 100-point sliding scale from “Not at all certain” to “Extremely certain.”

Participants then completed two comprehension checks about the determinism argument. The first measured bypass comprehension: “According to (the determinism) argument, do people’s desires, beliefs, and decisions have an effect on what they do?,” with response options “yes,” “no,” and “I don’t know,” the correct answer being “yes.” We then assessed otherwise comprehension: “According to (the determinism) argument, is it possible for a human to behave in any way other than they ultimately do?” with response options “yes,” “no,” and “I don’t know,” the correct answer being “no.” Participants then reported whether they personally agreed with each argument on a 100-point sliding scale from “Not at all” to “Entirely.” Last, participants completed demographic questions and an attention check.

### Results

#### Comprehension Checks

With this description of determinism, 67.9% of participants passed the bypass comprehension check and 67.1% passed the otherwise comprehension check. Note, these are still quite low considering the argument stated 1) “*People’s mental states (their beliefs, desires, and decisions) do have an effect on what they do…”* and the bypass comprehension question was “Do people’s desires, beliefs, and decisions have an effect on what they do?” and 2) “*No human is ever capable of having different thoughts or behaving in any way different than they ultimately do*” and the otherwise comprehension question was “is it possible for a human to behave in any way other than they ultimately do?”

**Table 4:** Compatibilist responses by order and moral conditions in Study 3a.

Order condition	Moral condition	Yes	No	Don’t know
Free will first	Moral	41.6%	56.2%	2.2%
	Not moral	33.7%	65.1%	1.2%
Free will second	Moral	20.7%	72.4%	6.9%
	Not moral	33.3%	61.9%	4.8%

**Table 5:** Compatibility results in Study 3a.

	Comparison	*OR*	*b*	*SE*	*Wald*	*p*	*95% CI*
Order condition	Yes vs. No	0.96	−0.04	0.33	0.01	0.905	0.51, 1.83
	Yes vs. DK	4.14	1.42	1.15	1.53	0.216	0.44, 39.39
Moral condition	Yes vs. No	0.53	−0.63	0.36	3.18	0.075	0.26, 1.07
	Yes vs. DK	0.43	−0.85	0.71	1.41	0.234	0.11, 1.73
Interaction	Yes vs. No	2.69	0.99	0.48	4.35	0.037	1.06, 6.83
	Yes vs. DK	1.49	0.40	1.44	0.08	0.782	0.09, 24.96

*‘DK’ stands for ‘Don’t Know’ response. ‘Yes’ response is the reference condition.*

#### Argument Compatibility

**FIGURE 3:**
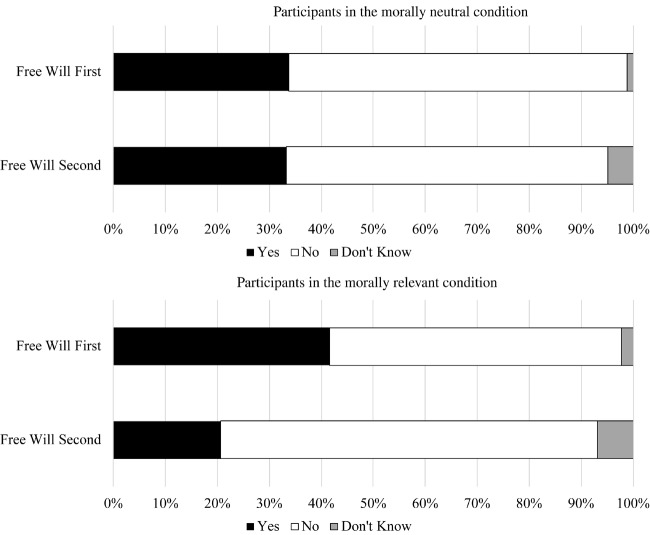
Compatibility responses by order condition within each moral condition in Study 3a.

A multinomial logistic regression tested whether the moral relevance condition moderated the influence of the order condition on compatibilist responses. As can be seen in Tables 4, 5 and Figure 3, the difference in the relative proportion of compatibilist responses to incompatibilist responses between the free will first and free will second argument conditions was significantly larger in the morally relevant condition than the morally neutral condition with a small to medium effect size. Specifically, in the morally neutral condition, participants were no more likely to be compatibilist when evaluating whether free will is compatible with determinism (free will second condition; 34.1%) than when evaluating whether determinism is compatible with free will (free will first condition; 35%), χ^2^ = 0.01, *p* = 0.905. However, in the morally relevant condition, participants were more compatibilist when evaluating whether determinism can be true if people have free will (42.5%) than when evaluating whether people can have free will if determinism is true (22.2%), χ^2^ = 7.86, *p* = 0.005.

### Discussion

Whether two arguments are compatible ought to be independent of which one is viewed first, but Study 3a found that the order made a difference, at least sometimes. When told to assume determinism was true, many people denied that free will could also exist. In contrast, when told to assume that people have free will, they were more compatibilist, allowing that determinism could also be true. Moreover, this pattern only held when people were contemplating a morally bad action (a robbery), and not when contemplating a morally neutral action (eating pasta). Past work has indicated that people are especially motivated to uphold belief in free will when contemplating morally bad actions. This motivational pattern extends to judgments about compatibilism, according to Study 3a’s findings.

These results suggest once again that folk intuitions about compatibilism are not reflective of some rational, coherent process. Rather, the moral implications of arguments and order of presentation of arguments influence these judgments. Consistent with predictions, argument order only mattered when the arguments had moral implications. However, within the morally relevant condition, the pattern of results was opposite to our predictions. We thought participants would embrace compatibilism after being asked to assume determinism is true, in order to preserve their belief in free will. Instead, they were more compatibilist after assuming people have free will and contemplating whether determinism could also be true. We could conclude that we had it backward, and that people are mainly motivated to preserve belief in determinism, but that seems intuitively implausible. Perhaps then those who were asked to assume free will and contemplate whether determinism could also be true were more compatibilist as a way of upholding belief in free will and responsibility. The reason for this motivated strategy would not be clear to us, but that is precisely the point. Participants may not be making these judgments in a rational fashion. They may simply respond in whatever way seems to them most likely to uphold their belief in free will and responsibility. Study 3b was designed as a follow-up to help interpret the findings of Study 3a.

## Study 3B

Rather than inferring (from inconsistent patterns of judgment) whether compatibilist responses reflect desires to uphold free will and moral responsibility, Study 3b directly asked participants whether these beliefs influenced their judgments. Study 3b was identical to Study 3a, except (1) it only included the morally relevant condition and (2) participants also rated the extent to which a variety of thoughts influenced their conclusion regarding whether the arguments were compatible. These thoughts included the assertions that free will is true, that humans are responsible, that determinism is false, or that their gut compelled them, among others. Recall that participants were instructed NOT to let their agreement with either argument influence their conclusion and simply to consider whether the two arguments were logically compatible. On that basis, considerations of whether free will is true should not have influenced their judgments.

Based on the results of Study 3a, in which participants who read the free will argument first were more compatibilist than those who read the free will argument second (in a morally relevant condition), we expected that these participants would be more likely to report that their belief in free will and responsibility influenced their conclusion. Though it is likely that individuals who are motivated to uphold free will and responsibility might also be motivated to deny determinism, it is also possible that motives to deny determinism might increase incompatibilist responding. Because of these competing motives, we did not have *a priori* predictions about whether individuals who read the free will argument first would be more likely to cite their disbelief in determinism as influencing their conclusion. We also did not have *a priori* predictions regarding the other reasons participants could report as influences on their judgment, but they may nonetheless be helpful for further understanding what kinds of considerations influence compatibilist judgments.

### Methods

The methods were nearly identical to Study 3a (*n* = 192 undergraduates at a northeastern United States university; 104 female, *M*_age_ = 19.41) except only the morally relevant condition (the robbery) was presented in Study 3b, and the certainty question was not asked. The only other difference was that after participants reported whether the second argument was compatible with the first, they read, “*We are interested in how you came to your conclusion on the previous page. Likely, you had many different thoughts and considerations that influenced your decision. Below is a list of various thoughts people have when considering such arguments. Please indicate the extent to which the thought influenced your conclusion.*” They then read twelve potential thoughts and rated the extent to which each thought influenced their conclusion on 9-point scales from “Did not influence my conclusion at all” to “Completely influenced my conclusion.” The potential thoughts were “Human thoughts and actions ARE/ARE NOT predetermined,” “Humans DO/DO NOT have free will,” “Humans ARE/ARE NOT responsible for their behavior,” “My gut says these arguments ARE/ARE NOT compatible,” “These arguments directly support/oppose each other,” “These arguments are unrelated to each other,” and “I do not understand these arguments.” Note that ten of the twelve potential thoughts were part of an opposing pair of two questions. For each pair, one item was reverse-scored and averaged with the opposing thought for a total of seven potential thoughts. There was also an open-ended box for other thoughts.

### Results

#### Comprehension Checks

63.5% of participants passed the bypass comprehension check and 76.6% passed the otherwise comprehension check.

**Table 6:** Compatibility results by order condition in Study 3b.

Compatibility response	*Free will first*	*Free will second*	*OR*	*b*	*SE*	*Wald*	*p*	*95% CI*
Yes	26.6%	21.4%						
No	69.1%	77.6%	1.39	0.33	0.34	0.94	0.332	0.71, 2.72
Don’t know	4.3%	1.0%	0.30	−1.21	1.16	1.10	0.295	0.03, 2.87

*‘Yes’ response is reference category.*

#### Argument Compatibility

**FIGURE 4:**
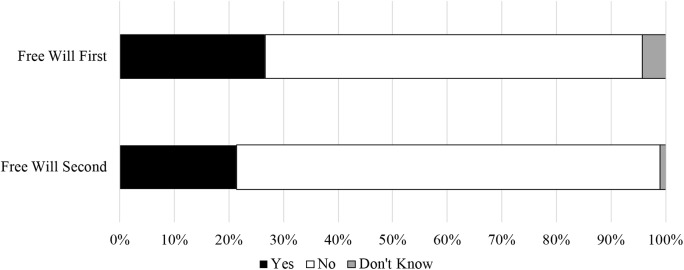
Compatibility responses by order condition in Study 3b.

Once again, the proportion of compatibilist (26.6%) to incompatibilist (69.1%) responses was higher among those who evaluated whether determinism was compatible with free will (free will first condition) relative to those who evaluated whether free will is compatible with determinism (free will second condition; 21.4% vs. 77.6%). However, this effect was small, *OR* = 1.39, and not statistically significant, *p* = 0.332 (see Table 6 and Figure 4).

#### Mini Meta-Analysis

Because the argument compatibility result did not reach statistical significance, but the independent and dependent variables were identical in Studies 3a and 3b, we conducted a fixed effects meta-analysis (see Hedges and Vevea, 1998; Valentine et al., 2010) of this outcome variable in Studies 3a and 3b. We computed an *r* effect size from the odds ratios, which were converted to Zrs, weighted by *n*–3, averaged, and then converted back to *r* (Goh et al., 2016). We used Stouffer’s *Z*-test to estimate statistical significance. This revealed a small, marginal effect overall, *r* = 0.14, *p* = 0.058^7^.

Due to the partial replication failure of Study 3b, these results should be interpreted with caution. However, the results of the meta-analysis suggest that there is a small effect of the order condition such that there was generally a higher proportion of compatibilist to incompatibilist responses when participants read the free will argument before the determinism argument than vice versa.

#### Reasons for Argument Compatibility

We conducted independent samples *t*-tests on each thought participants could cite as influencing their judgments (see Table 7). The results showed that relative to participants who read the free will argument second, participants who read the free will argument first (the more compatibilist group) were more likely to report that their beliefs that people have free will and that determinism is false influenced their conclusion, and marginally more likely to report that their beliefs that people are responsible for their behavior influenced their conclusion. They were also more likely to report that their gut feeling that the arguments were compatible influenced their conclusion. The reasons that did not significantly differ between conditions were the extent to which they did not understand the arguments, and the two (arguably) rational responses: the extent to which the arguments supported or opposed each other and the extent to which the arguments were unrelated.

We conducted additional independent samples *t*-tests on each of these thoughts between those who were compatibilist vs. incompatibilist (collapsed across the two conditions). Compatibilists were more likely than incompatibilists to report that their beliefs that people have free will and that people are responsible for their behavior influenced their conclusion. There was a marginal tendency for compatibilists to report that their inability to understand the arguments influenced their conclusion. In contrast, incompatibilists were more likely to report that their belief that determinism is false influenced their conclusion than compatibilists. Incompatibilists were also more likely to report that their belief that the arguments opposed each other influenced their conclusion than compatibilists were to report that their belief that the arguments supported each other influenced their conclusion,^8^ though of course, the arguments opposing each other is a rational reason for incompatibilism whereas the arguments supporting each other would not be necessary for compatibilism. Compatibilists and incompatibilists did not differ in the extent to which their belief that the arguments were unrelated influenced their conclusion. This is perhaps surprising because it would be a rational reason for a compatibilist response. Last, they did not differ in their reports that their gut feelings influenced their conclusion, though both groups were above the midpoint.

**Table 7:** Reasons for argument compatibility by condition and response in Study 3b.

Reasons	*Free will first*		*Free will second*					
	*M*	*SD*	*M*	*SD*	*t*	*df*	*p*	*95% CI*
Free will	5.46	1.21	4.78	1.64	−3.24	190	0.001	−1.09, −0.27
Responsibility	5.28	1.29	4.87	1.68	−1.91	190	0.058	−0.84, 0.01
Determinism	4.45	1.43	3.88	1.69	−2.51	190	0.013	−1.02, −0.12
Gut	3.49	1.79	2.96	1.69	−2.09	190	0.038	−1.02, −0.03
Oppose	5.21	1.39	5.55	1.53	1.60	190	0.111	−0.08, 0.75
Unrelated	2.59	1.75	2.60	1.75	0.07	190	0.947	−0.48, 0.52
Understand	2.02	1.54	1.79	1.36	−1.12	190	0.262	−0.65, 0.18

	***Compatibilists***		***Incompatibilists***					

Free will	5.59	1.25	4.99	1.53	2.38	185	0.018	0.10, 1.09
Responsibility	5.59	1.21	4.93	1.59	2.58	185	0.011	0.15, 1.16
Determinism	3.42	1.43	4.40	1.60	−3.68	185	<0.001	−1.50, −0.45
Gut	4.57	1.93	4.99	1.90	−1.30	185	0.196	−1.06, 0.22
Oppose	3.37	1.66	5.72	1.50	−8.97	185	<0.001	−2.86, −1.83
Unrelated	2.65	1.84	2.54	1.73	0.38	185	0.705	−0.48, 0.70
Understand	2.17	1.66	1.74	1.31	1.83	185	0.069	−0.03, 0.91

*Interpretations of reasons (i.e., the directions of the assertions) are described in text.*

### Discussion

As in Study 3a, participants were generally more compatibilist when they read the free will argument first (though the pattern was weaker in strength and short of significance) than when they read the determinism argument first. Participants who read the free will argument first (the more compatibilist group) were also more likely to report that their beliefs in free will and responsibility influenced their conclusion than those who read the free will argument second. All compatibilists (regardless of experimental condition) were also more likely than incompatibilists to report that their beliefs in free will and responsibility influenced their conclusion. This provides the most direct support of our hypothesis that motives to uphold free will and moral responsibility influence compatibilist judgments.

Participants had been instructed not to let their beliefs influence their judgments and yet openly admitted to doing so. On the 7-point scale, a mean of 1.00 would indicate that their beliefs about free will and responsibility did not influence their conclusions, but the averages on these two items were above 5.00, and over 90% reported that their beliefs in free will and responsibility influenced their conclusion at least somewhat. Even independent of experimental manipulations, it seems the majority of people are unable to make judgments about compatibilism without considering their belief that people have free will and are responsible for their behavior (or at minimum, people think these beliefs influenced their conclusions).

Participants who read the free will argument first (the more compatibilist group) were also more likely than others to report that their disbelief in determinism influenced their conclusion. This is difficult to interpret because incompatibilists as a group were more likely to cite their disbelief in determinism as an influence on their conclusion than compatibilists. This could mean there are competing motives such that motives to deny determinism increase incompatibilist responding, but motives to maintain free will and responsibility (which may increase compatibilist responding) might also increase motives to deny determinism.

What other considerations influence compatibilist judgments? Compatibilists and incompatibilists both reported being highly influenced by their gut feelings. Compatibilists were more likely to say that their inability to understand the arguments influenced their conclusion [perhaps consistent with work showing greater incompatibilism among those with more knowledge of the free will debate (Schulz et al., 2011)]. Even after omitting participants who responded that they did not know whether the arguments were compatible or incompatible, 33% reported that their inability to understand the arguments influenced their conclusion at least somewhat. This may mean that up to 33% of participants who provided compatibilist or incompatibilist judgments simply guessed despite having been provided an “I don’t know” option.

The results of this study suggest that the order in which arguments are presented might activate different motives, which may then influence compatibilist judgments. The results also suggest that participants may be somewhat self-aware of their own motivations and reasons for drawing conclusions about compatibilism, which include their beliefs about free will, responsibility, determinism, their gut feelings, and even their inability to understand the arguments, in addition to more rational reasons such as whether the arguments contradict each other.

## Study 4

Study 4 sought to test whether motives to maintain moral responsibility would lead to inconsistent compatibilist judgments even *within* subjects. Participants read a determinism passage and evaluated whether free will and moral responsibility were compatible with that passage. Participants then read a passage that argued that people should be held morally responsible regardless of whether they have free will due to the deterrent efficacy of punishment. The argument said nothing about whether people actually have free will, but it removes one motivation to assert free will, namely its ostensible importance for preserving moral responsibility. Participants then responded to the same free will and moral responsibility questions as before. We expected that participants would reduce free will compatibilism, but maintain moral responsibility compatibilism. These results would suggest that people are less inclined to assert free will compatibilism when free will is less necessary for preserving moral responsibility, even though the only new information they received was irrelevant to the actual truth of free will or compatibilism.

It is challenging to measure bias in a within-subjects design. People feel compelled to maintain consistency in their judgments, and insofar as they are reluctant to answer the same question in different ways, the design may preclude finding differences (see Uhlmann et al., 2009; Crawford et al., 2015; Winegard et al., 2018). Nonetheless, evidence of motivated change in judgments would contribute to the case that people furnish responses to free will questions partly to uphold moral responsibility.

### Methods

This study was preregistered for sample size, analyses, and predictions (https://aspredicted.org/6kj35.pdf). In an online study, 162 undergraduates at a southeastern United States university (107 female, *M*_age_ = 18.78)^9^ were informed that they would read two passages, and alternate responding to each. Participants first read a determinism passage, which was adapted to combine elements from Nahmias et al. (2005, 2006) and Nichols and Knobe (2007) descriptions, “*Everything that happens in the universe is completely caused by what happened before it… If the universe was re-created over and over again… this would cause the exact same events… people’s mental states are part of the causal chains that lead to their actions… For example, 1 day John decided to rob a convenience store… it had to happen that John would decide to rob that convenience store.”*

Participants were asked to assume the passage was correct while responding to two questions, “If the argument is correct, can people have free will?” and “If the argument is correct, can people be held morally responsible?” on 7-point scales from “Definitely no” to “Definitely yes.” Participants were then directed to a second passage adapted from Clark et al. (2017), which argued that free will is unnecessary for moral responsibility, “*…recently, philosophers and scientists are realizing that free will is not a requirement for holding people morally responsible… If humans know that bad behavior will be punished, they will be less likely to perform that bad behavior. Holding people morally responsible is not an issue of free will; it is merely a means of discouraging behavior destructive to society.”*

Participants were then asked two questions about whether the argument supports or opposes (1) the existence of human free will and (2) human moral responsibility, with response options that it supports, opposes, or does neither. For purposes of reducing demand characteristics, participants were told that they would be responding to more questions about the second argument, but that first they would respond to a few more questions about the first argument, some familiar and some new. Participants then re-read the first argument and responded to the same free will and moral responsibility questions as before. Participants also completed two comprehension checks measuring bypass and otherwise comprehension, “According to this argument, do people’s beliefs and desires have an effect on what they do?” and “According to this argument, given the laws of nature and the initial conditions of the universe, do later events (including human decisions) have to happen as they do?” the correct answer being “yes” for both questions.

For purposes of upholding the cover story, participants were then asked to describe the second argument in their own words. Participants also answered a number of demographic questions and completed an attention check.

### Results

In this study, 58.0% of participants passed the bypass comprehension check, and 76.5% of participants passed the otherwise comprehension check. A slim majority of participants (52.5%) correctly reported that the argument that opposed the necessity of free will for moral responsibility neither opposed or supported the existence of human free will, and a large majority (75.3%) correctly reported that the argument supported human moral responsibility.

Two paired samples *t*-tests were conducted to determine whether reducing the necessity of free will in order to maintain moral responsibility would decrease reports that free will was compatible with determinism, but have no effect on reports of whether moral responsibility was compatible with determinism. There was a small (marginal) effect such that participants reported that free will was less compatible with determinism after reading that free will is unnecessary for moral responsibility (*M* = 3.97, *SD* = 2.11) than before (*M* = 3.76, *SD* = 2.02), *t*(161) = 1.66, *p* = 0.098, Cohen’s *d* = 0.131, whereas there was virtually no effect on moral responsibility judgments (Time 1 *M* = 4.41, *SD* = 2.12, Time 2 *M* = 4.33, *SD* = 1.94), *t*(161) = 0.57, *p* = 0.570, Cohen’s *d* = 0.049.

### Discussion

After considering the argument that free will is not necessary for preserving moral responsibility, participants reduced their reports that free will is compatible with determinism. This effect was small and did not reach traditional levels of statistical significance. Still, the expected pattern emerged with a within-subjects design, in which consistency pressures would likely attenuate any effect. Despite this limitation, Study 4 replicated the basic pattern found in Studies 1–3b, that judgments about free will and compatibilism are influenced by desires to preserve important beliefs, in this case, that people can be held morally responsible.

## Study 5

In Studies 1–3b, compatibilist responses among the folk ranged from 20.7 to 59.3%. Moreover, assertions of compatibilism varied as a function of mere consideration of the truth of determinism, the moral implications of arguments, the order of presentation of arguments, and explicit beliefs about free will and moral responsibility. None of these is logically relevant to the possible truth of compatibilism. Overall then, it seems people are evaluating these arguments in ways that favor their preferred beliefs about reality. To illustrate this point, we turned to a more extreme example.

Most compatibilist philosophers do not posit that compatibilist free will entails the existence of immaterial souls (e.g., Monroe and Malle, 2014) or that immaterial souls are compatible with scientific determinism. Rather, compatibilist philosophers have defined free will as the capacity to make rational choices in the absence of coercion, an ability consistent with a modern, scientific understanding of the human brain.

To our knowledge, very few scholars these days believe in the existence of immaterial souls. The United States population, however, is quite religious, largely Christian, and likely does maintain fairly strong belief in immaterial souls (64% believe souls survive after death; The Harris Poll, 2013). Therefore, the folk might be more than willing to assert that immaterial souls are compatible with scientific determinism in order to maintain their belief that souls exist. Study 5 tested whether people find immaterial souls compatible with scientific determinism (henceforth referred to as soul compatibilism). Study 5 also tested whether those who believe in immaterial souls and those who believe in determinism would be especially likely to report that immaterial souls and scientific determinism are compatible. If immaterial soul believers were more soul compatibilist, it would demonstrate once again that people evaluate argument compatibility in a way that allows them to maintain their prior beliefs. If determinism believers are also more compatibilist, it would provide additional support for Nichols (2007) suggestion that compatibilism is particularly attractive for those who believe in determinism, because it allows them to maintain other important beliefs (free will, moral responsibility, and perhaps immaterial souls).

### Methods

Adults (*n* = 101; 46 female, *M*_age_ = 33.50) participated on Amazon mechanical turk (MTurk) in exchange for a small payment^10^. Participants were told they were going to evaluate the logical compatibility of two arguments written by two different professors at prominent universities. They were asked to read one argument, assume it was true (regardless of whether they personally agreed it was true), and then evaluate whether a second argument could then also be true. The first argument supported the truth of determinism, “… *everything that happens is completely caused by whatever happened before it… mental states are part of the causal chains… Given that the past happened the way it did, all human decisions ever made had to happen the way they did.”* This description was written to combine elements from Nichols and Knobe (2007) and Murray and Nahmias (2014). The second argued that people have immaterial souls, “…*human experience cannot be reduced to purely physical causes… The human individual is part physical body, part immaterial soul. When people die, the body continues to physically exist, but your soul is no longer present in the body. This soul is the source of each individual’s unique, subjective experience of the world…”* This second description was written by the authors.

Participants were then asked whether the immaterial soul argument could possibly be true if they assumed the determinism argument was true (regardless of whether they personally agreed), on a 100-point sliding scale from “Definitely no” to “Definitely yes.” Participants were then asked to explain in a few sentences how they came to their conclusion.

Participants then completed a measure of bypass comprehension: “According to (the determinism) argument, do people’s desires, beliefs, and decisions have an effect on what they do?,” with response options “yes,” “no,” and “I don’t know,” the correct answer being “yes,” and a measure of otherwise comprehension: “According to (the determinism) argument, is it possible for a human to behave in any way other than they ultimately do?” with response options “yes,” “no,” and “I don’t know,” the correct answer being “no.” Participants then reported whether they personally agreed with each argument on 0 (Not at all) to 100 (Entirely) sliding scales. Last, they completed a variety of demographics, suspicion probes, and an attention check.

### Results

#### Descriptives

Consistent with expectations, participants generally did not believe in scientific determinism (35.6% of participants above the midpoint; *M* = 41.44, *SD* = 31.21), and were significantly below the midpoint, *t*(100) = −2.91, *p* = 0.004. Also as expected, participants generally did believe in immaterial souls (64.4% of participants above the midpoint; *M* = 61.62, *SD* = 30.59), and were significantly above the midpoint, *t*(100) = 3.65, *p* < 0.001.

#### Comprehension Check

With this description of determinism, 85.1% of participants comprehended that determinism does not entail bypassing. And participants did reasonably well comprehending that determinism means that people cannot behave other than they ultimately do, with 74.3% passing the otherwise comprehension check.

#### Argument Compatibility

A single sample *t*-test revealed that people generally do believe that immaterial souls are compatible with scientific determinism (*M* = 60.64, *SD* = 30.34, 65.3% of participants above the midpoint), which was statistically different from zero, *t*(100) = 20.09, *p* < 0.001, and significantly above the midpoint, *t*(100) = 3.36, *p* = 0.001. Furthermore, stronger belief in souls (*r* = 0.37, *p <* 0*.001*) and stronger belief in determinism (*r* = 0.26, *p* = 0*.009*) were associated with stronger soul compatibilism.

### Discussion

The majority of laypersons in our sample believed immaterial souls were compatible with scientific determinism. This sample was recruited from a widely used online population, and showed evidence of relatively high conceptual sophistication, specifically by doing better than our previous samples on the comprehension checks. Even so, those who believed in souls and those who believed in determinism were more likely to agree that they were compatible^11^. These support the hypothesis that compatibility judgments are influenced by personal motivations. The more people want to believe in either souls or determinism, the more they seek to reconcile that belief with other assertions.

To our knowledge, most compatibilist philosophers who promote the idea that the folk are firmly in agreement with them do not posit that immaterial souls are compatible with deterministic universes. When it comes to issues of free will, souls, religion, and the like, the opinion of the majority perhaps should not be considered a legitimate source of evidence about reality.

## Study 6

Of course, regardless of whether compatibilist philosophers would disagree with the folk that immaterial souls are compatible with scientific determinism, we cannot say that such beliefs are necessarily irrational. Study 6 sought to demonstrate that people evaluate new information in an irrational, incoherent fashion that favors the existence of human free will. We presented participants with an argument about primate free will and manipulated its implications for human free will. In one condition, participants were told that if primates have free will, then humans do not have the kind of free will that makes them personally responsible for their behavior; in the other, that they do. Participants then reported their belief in primate free will. If humans seek only to uphold human free will, they will agree that primates have free will when it indicates that humans also have free will, but they will disagree that primates have free will if it diminishes human free will.

### Methods

MTurk adults (*n* = 201; 88 female, *M*_age_ = 34.39) participated in exchange for a small payment. Participants were told they would read and evaluate a passage written by a prominent biology professor (that was actually written by the present authors) and were randomly assigned to one of two conditions. The details of the argument were held constant across two conditions, but the conclusion was manipulated to indicate that if primates have free will, the free will that makes humans personally responsible for their behavior either does or does not exist.

#### Primate Free Will Argument

When it comes to decision-making, human beings and other primates (e.g., other great apes, monkeys) have a great deal in common. Studies suggest that chimpanzees do show some degree of self-awareness and can anticipate how their actions will impact the environment around them. While chimpanzees and other great apes do show some capacity to reason, their ability to understand cause-and-effect relationships approximates that of human toddlers. This all raises the question of whether chimpanzees and other primates have “free will.” In considering the similarities and differences in cognitive functioning between humans and other primates, we must conclude that if other primates have free will, then the free will that makes people personally responsible for their behavior does (not really/indeed) exist.

Participants were then asked whether they believe other primates have free will, whether the arguments support the conclusion that other primates have free will, whether they believe humans have free will, and whether the arguments support the conclusion that humans have free will on 100-point sliding scales from “Definitely no” to “Definitely yes.” Last, participants completed demographics, suspicion probes, and an attention check.

### Results

We conducted a multivariate analysis of variance (MANOVA) on the four outcome measures. Participants who read that primate free will supports human free will and responsibility were more likely to agree that primates have free will (*M* = 68.89, *SD* = 25.67) and that the arguments supported the conclusion that primates have free will (*M* = 69.65, *SD* = 22.53), than participants who read that primate free will opposes human responsibility (*M* = 56.51, *SD* = 29.24, *M* = 49.42, *SD* = 28.01, respectively), *F*(1,199) = 10.21, *p* = 0.002, $\eta_{p}^{2}$ = 0.05, and *F*(1,199) = 32.00, *p* < 0.001, $\eta_{p}^{2}$ = 0.14, with medium to large effect sizes^12^.

Participants who read that primate free will supports human responsibility were also far more likely to agree that the argument supported the conclusion that humans have free will (*M* = 82.51, *SD* = 21.03), than participants who read that primate free will opposes human responsibility (*M* = 57.31, *SD* = 34.92), *F*(1,199) = 38.84, *p* < 0.001, with a large effect size, $\eta_{p}^{2}$ = 0.16. However, belief in human free will was virtually identical (and very high) regardless of whether the existence of primate free will indicated human responsibility (*M* = 87.75, *SD* = 18.93) or a lack of human responsibility (*M* = 87.58, *SD* = 19.80), *F*(1,199) = 0.00, *p* = 0.952, $\eta_{p}^{2}$ = 0.00.

### Discussion

Study 6 provided further evidence that layperson judgments about free will are subject to motivated bias. All participants read exactly the same account of how non-human primates can think and reason. The only difference was one to two words in the final sentence, in which the author extrapolated the implications for humankind: the primate evidence supported the conclusions that humans do or do not have free will and moral responsibility. Minimizing the plausibility that participant responses reflect normative reasoning, the information relevant to the main dependent variable (whether primates have free will) was identical in both conditions (i.e., both conditions described identical reasoning capacities for primates). The manipulation only regarded the implications for human free will. Normative reasoning counterexplanations are minimized to the extent that the main dependent measures regard the *identical* information rather than the manipulated information (Ditto et al., 2018b; though also see Ditto et al., 2018a for a discussion of the challenges of ruling out normative counterexplanations completely). So, Study 6 supports the contention that people are motivated to preserve free will.

Participants’ answers seemed designed to preserve their own belief in free will. First, participants’ belief in human free will was uniformly high regardless of what they read about primates and its implications for humankind. Second, they thought the essay supported the existence of primate free will more when it meant humans have free will than when it meant humans did not. Third, their own private opinions about primates changed in the same way: They agreed that primates have free will mainly when primate free will supported human free will and responsibility. Once more, people appear incapable of evaluating information that has implications for the existence of human free will in a rational manner and rather evaluate information a way that allows them to assert its truth.

## General Discussion

Whether everyday people believe in free will has been the subject of a fair amount of study. The answer appears to be a resounding yes. Moreover, variations in the extent of this belief have been shown to predict a wide range of attitudes and actions (e.g., Baumeister et al., 2009; Baumeister and Brewer, 2012; Clark et al., 2017). That this belief influences subsequent attitudes and behaviors seems to suggest that people not only have opinions about free will but also that these opinions are well thought out. This prompted experimental philosophers to ask how laypeople think about the same dilemmas that philosophers contemplate, such as whether free will is compatible with a deterministic worldview. However, different researchers have reached widely different conclusions about this, and more than a decade after this research began, a consensus has not been reached.

The present results help clarify some of the seemingly contradictory findings in the compatibilism literature. For example, they provide nuance to Nichols and Knobe (2007) findings that affective reactions to specific stories about immoral behavior increase compatibilist responding and the seemingly contradictory findings by Feltz et al. (2009), Feltz and Millan (2013), and Feltz and Cova (2014) that affective reactions do not influence compatibilist responding. Specifically, Feltz and colleagues manipulated affect using only morally bad scenarios and did not manipulate motivations to hold a specific morally bad actor responsible (as Nichols and Knobe did, though this was not their exact intended manipulation). If motivations to uphold moral responsibility increase compatibilist responding, it is not surprising that Feltz and colleagues found high rates of compatibilism in both high and low affect *morally bad* conditions and that Nichols and Knobe found high rates of compatibilism in their morally bad condition but not in their abstract moral responsibility condition (because exposure to morally bad actions increases desires for moral responsibility). Thus, the present results suggest that it is not affect *per se* that increases compatibilist responding but rather desires to uphold human moral responsibility. This also explains why Roskies and Nichols (2008) found that people were more compatibilist about their own universe, and why Nahmias et al. (2005) found that people refused to deny the ability to choose to an immoral actor–people seek to uphold the ability to hold fellow human beings morally responsible, and particularly for morally bad actions.

### The Motivation Problem

The present findings suggest that people’s responses to these abstract dilemmas and questions are often driven by motivations to sustain their favored beliefs–in particular their belief that human beings have free will and deserve to be held morally responsible for their actions. In Study 1, participants who were asked to consider the possibility that our universe is deterministic were more compatibilist than those not asked to consider this possibility. In principle, whether our own universe happens to be deterministic should be irrelevant to whether determinism and free will can co-occur. As with Nichols (2007) suggestion that compatibilism emerged among philosophers who came to accept determinism, so the folk might similarly find compatibilism compelling when determinism poses potential threats to moral responsibility.

The motivation to preserve moral responsibility was apparent in all of the present studies. In Study 2, participants who considered concrete instances of moral behavior were more likely to report that compatibilist (and libertarian) free will were sufficient for moral responsibility than participants who were asked about moral responsibility more generally. In Study 3a, the order in which participants read free will and determinism arguments influenced their reports of whether the two arguments were compatible–and only when the arguments had moral significance. Participants were more likely to report that determinism was compatible with free will (i.e., the free will argument came first) than that free will was compatible with determinism (determinism argument came first). In Study 3b, participants who read the free will argument first were particularly likely to confess that their beliefs in free will and moral responsibility and their disbelief in determinism influenced their conclusion, despite that they were instructed to disregard their own beliefs. The compatibility of two positions should be directionally symmetrical, but it appears the order of arguments influenced participants’ compatibilist judgments as well as the extent to which participants allowed their prior beliefs to influence their conclusions.

In Study 4, participants reduced their belief that free will is compatible with determinism after reading a passage that argued that free will is unnecessary for moral responsibility. Thus, when free will was no longer needed to preserve moral responsibility, participants were less inclined to resolve any conflict between free will and determinism.

Studies 5 and 6 did not directly address folk compatibilism but demonstrated in other ways that people’s judgments are biased toward preserving their beliefs about characteristics of human responsibility. In Study 5, the majority of participants found immaterial souls compatible with a scientifically deterministic universe, to the strongest degree when they believed in immaterial souls or scientific determinism. In Study 6, participants who read identical information about the capacities of primates were far more likely to agree that primates have free will if primate free will supported rather than opposed human free will and responsibility. Thus, even when provided identical information, participants evaluated information in a way that favored the existence of human free will and moral responsibility.

### The Comprehension Problem

Complicating matters further, participants had a very difficult time comprehending deterministic descriptions and compatibilist free will. In Study 3b, 33% of participants who rendered a judgment (i.e., omitting participants who responded that they did not know whether free will and determinism were compatible) openly admitted that their inability to understand the arguments influenced their conclusion at least somewhat. Across all of our studies, approximately 15–42% of participants did not comprehend that compatibilist free will does not entail bypassing. (That is, they did not understand that compatibilist free will recognizes a person’s thoughts and intentions as part of what causes behavior.) One could argue that we did a poor job describing compatibilist free will, but this failure rate is comparable to Murray and Nahmias (2014) paper, in which the main goal was to eliminate this problem. In their Study 2, they added language to a variety of deterministic descriptions explicitly stating that determinism does not entail bypassing, and still found failure rates from 12 to 49%. Thus, the present studies are at least on par with even the best of the work currently available.

Even more challenging was getting participants to comprehend that determinism precludes the ability to do otherwise given a particular chain of past causes. Across our studies, participants failed this comprehension check approximately 23–65% of the time. It is not entirely clear how typical this failure rate is. Very few studies report comprehension failures for the ability to do otherwise given that the past happened as it did and the natural laws are as they are. In Nichols and Knobe (2007) Study 2, approximately 32% of participants failed to comprehend that in principle, in a deterministic universe, everything that happens is predictable, but they note there are problems interpreting this failure rate. Feltz and Millan (2013) reported 20–36% comprehension failure rates, but these figures combined failures to comprehend determinism and failures to comprehend fatalism. Nahmias et al. (2005) included questions about whether actors in a deterministic universe had the ability to choose not to do what they did; 67% said yes when the actor robbed a bank, whereas 45% said yes when the actor went jogging, and 35% said yes when the actor saved a child. Whether these are comprehension failures is up for debate, but at least they suggest that how people comprehend deterministic scenarios appear vulnerable to moral responsibility motives as well. In addition to the problem of moral responsibility motives, it is clear that the folk struggle a great deal with understanding compatibilist free will and determinism in the way philosophers do. And to our knowledge, nobody has successfully dealt with this issue (ourselves included).

It is possible that everyday people simply cannot understand the nuances of these crucial concepts. Given this challenge, it is critical that future research on folk compatibilism includes appropriate comprehension questions and reports these results, practices that are currently rare in the field.

### Quibbles and Qualms

Some compatibilists may quibble with some of our descriptions of determinism, perhaps especially the descriptions asserting that in deterministic universes, people “lack the capacity to choose among alternative courses of action” (or similar). Many incompatibilists would accept this description, but some compatibilists would argue that it is misleading. Even in a deterministic universe, people have the capacity to do otherwise in the sense that they could have done otherwise if something about the past or laws of nature had been otherwise (e.g., Vihvelin, 2004; Turner and Nahmias, 2006). In other words, had the initial conditions of the universe or the laws of nature been different, an individual could have done something different from what they did in the present universe. Therefore, they *do* have the capacity to do otherwise.

One could reasonably speculate that when regular people think about the ability to do otherwise, they do not think their ability to do otherwise is constrained to alternate relevant possible worlds with different natural laws. In fact, motives to hold individuals morally responsible may influence whether people believe the ability to do otherwise is possible in a deterministic universe (Nahmias et al., 2005). Thus, the issue of whether the folk believe that the ability to do otherwise is possible in a deterministic universe (like many compatibilist philosophers) may be vulnerable to the same motivations as their evaluations of whether moral responsibility is possible with this limited variety of the ability to do otherwise.

As experimental philosophers could attest, designing studies with descriptions of determinism that satisfy all perspectives is challenging. We sought to avoid taking sides on the alternative universe issue, particularly because we suspect that such subtleties did not much preoccupy our research participants. Hence our procedures across multiple studies were designed to cater variably to the different perspectives. First, we used multiple descriptions of determinism across our studies and different comprehension checks that varied in multiple ways, including in their charitability to compatibilist vs. incompatibilist philosophers. For example, some of our studies stipulated that in a deterministic universe, a person would make a particular decision *only if specific events occurred* (Study 1), *given the initial conditions of the universe and the laws of nature* (Study 4), and *given that the past happened the way it did* (Study 6). Hopefully, these satisfy compatibilists. Second, we were careful to describe determinism in such a way that does not entail the problem of bypassing (e.g., “people’s mental states do have an effect on what they do,” “humans do have the capacity to act in accordance with their own thoughts and desires”), another distinction that is important to compatibilists.

### Summary and Suggestions for Future Work

We found variation in compatibilist responding depending on the moral relevance of arguments, the order of arguments, exposure to deterministic arguments, and agreement with arguments. But the present work is far from the first to note that framing effects can influence compatibilist responding. Roskies and Nichols (2008) take an optimistic view on the matter. They argue that the patterns of judgment are not random and incoherent and that we can make predictions about when the folk will be compatibilist. One might then consider what the optimal conditions are for assessing whether the folk are generally intuitive compatibilists. That is, under what conditions are the folk’s intuitions useful for deciding whether the burden of proof falls on compatibilists or incompatibilists to make their case for moral responsibility or a lack thereof? It seems philosophers go through painstaking efforts to get the folk to make these judgments in a sober, reflective, cognitively effortful manner to understand the *true* nature of their intuitions. They attempt to get the folk to approach the issue the same way they do.

In the real world, however, people typically hold others morally responsible when there has been a moral infraction. Then it might be cases in which people are considering concrete instances of morally bad behavior that have the most relevance to reality, even if in such cases people are the most likely to disregard reason and hold people morally responsible regardless of mitigating circumstances.

People do believe strongly in free will, and these beliefs affect their behavior in a variety of ways. These beliefs are not trivial and should be studied. However, the folk likely are not concerned with the metaphysical requirements for moral responsibility and instead simply care about holding people morally responsible when it is desirable and not manifestly unreasonable to do so. Indeed, holding fellow human beings morally responsible for their misbehavior appears crucial for living in social groups (e.g., Clutton-Brock and Parker, 1995; Henrich et al., 2006, 2010)–and this is true regardless of how one defines free will, and regardless of whether people have it. This likely explains why people seem to have *free-will-no-matter-what* intuitions (Feltz and Millan, 2013) and non-negotiable beliefs in human choice and control (Nahmias and Thompson, 2014).

It may not be fruitful or even coherent to assert definitively that folk have either compatibilist or incompatibilist intuitions (e.g., Feltz and Cokely, 2009; Schulz et al., 2011). They probably have both (Deery et al., 2014) and may not regard them as inconsistent with each other. For comparison, consider religious adherents. They often assert that they believe in official doctrine, e.g., that God can see all things at once, but when tested in the laboratory, they appear to find it more intuitive that God must visually scan one thing *before* seeing another and thus perceives more like a human than an omnipotent and omniscient entity. Barrett (1999) termed this Theological incorrectness, and contended that religious folk simultaneously hold multiple, sometimes contradictory, representations about religious entities. Intuitively, folk probably default to a rather human-like representation of God; however, they may use a different representation when deliberating about God in a theology seminar. The religious representation is impelled by the demands of the situation. The same probably holds for free will. People have multiple representations, some which possibly contradict each other.

Free will belief may stem largely from an intuitive feeling that one acts freely (Wegner, 2002), and applying this intuition to everyday life is likely far less cognitively demanding than logically evaluating the compatibility of free will with complex artificial universes. Like soul mates, souls, fate, and other abstract metaphysical concepts, people likely do not have consistent, coherent notions of free will, and they are likely incapable of reasoning about these concepts in the way philosophers would like them to.

We are optimistic about the project of experimental philosophy. However, we believe that for understanding how ordinary people represent abstruse concepts such as free will, the laboratory is likely to be uninformative, perhaps even confusing. This does not mean that the general goal of understanding what the folk consider necessary for moral judgments and what they mean when they use the terms “free” and free will” is misguided. After all there are billions of people on the planet and very few of them are philosophers. Folk beliefs about free will are much more consequential than the views of a small fraction of the population with degrees in philosophy. The error is not the pursuit of understanding how the folk use the word “free will,” but rather the expectation, made by some, that they use the word rationally or systematically.

We believe, along with others in the experimental philosophy tradition (e.g., Knobe and Nichols, 2008; Hansen, 2014), that an analysis of ordinary language usage would be more edifying. We can imagine, for example, a kind of philosophical ethnography that describes and analyzes the way concepts such as free will are used and function during conversation and debate and also in newspaper articles and blogs that are aimed at intelligent laypeople (Sytsma and Buckwalter, 2016). This would allow purchase on the many (and possibly contradictory) ways in which the terms “free” and “free will” are used in ordinary conversation, allowing philosophers and other analysts a glimpse into the folk mind without the unrealistic expectation that the folk use the term consistently or that they have only one representation for “free will.”

Perhaps in free flowing, intellectually undemanding conversations, for example, the folk generally use “free will” to mean “acted in accordance with desire without external constraint.” But in more intellectual conversations, conversations about higher level concepts, the folk use “free will” to mean, “acted without *internal* or external cause.” Then philosophers could see if the folk, in everyday intellectual conversations, seem to believe that free will is compatible with scientific determinism. This, we believe, would be a more genuine approximation of folk beliefs because it would illustrate how they think about the concept when *not compelled to accept (or not) scientific determinism by a vignette on a computer for an experiment.* Do the folk discount talk about determinism in everyday discourse? Do they argue against it in favor of free will? Or do they seem to talk about free will in a way that is consistent with scientific determinism? (Again, keeping in mind that they likely have more than one representation that corresponds to “free will”).

In other words, philosophers should get out of the lab and into the wild.

## Conclusion

Beliefs in free will probably arise for myriad reasons. Many philosophers who have explored the free will intuitions of the folk have tried to ascertain whether the folk are compatibilists or incompatibilists, that is, whether they believe that free will can be reconciled with a deterministic universe. But this is an abstruse philosophical question, one that even highly educated philosophers stumble over. Our findings suggest that researchers should be alert to the motivations, inconsistencies, misunderstandings, and other inadequacies in the answers people furnish regarding philosophical dilemmas. Everyday people may not be capable of furnishing meaningful and coherent conclusions about such challenging philosophical questions. Rather, they are content with the conclusion that people have free will, regardless of what it is, and that people who commit moral transgressions should be punished for failing to exercise that free will properly. These motives to uphold free will and moral responsibility may explain many of the discrepant findings in the experimental philosophy literature. Judgments with significance to human moral responsibility (e.g., judgments about human behavior, and particularly harmful human behavior) will motivate individuals to accommodate their desires to uphold human free will and moral responsibility–regardless of experimentally imposed constraints.

## Data Availability Statement

The raw data supporting the conclusions of this manuscript will be made available, without undue reservation, to any qualified researcher. They will also be publicly available on the lead researcher’s researchgate page: https://www.researchgate.net/profile/Cory_Clark.

## Ethics Statement

The data reported in this paper were collected in a manner consistent with ethical standards for the treatment of human subjects. The procedures were approved by the University of California, Irvine, the University at Buffalo SUNY, and the Florida State University Institutional Review Boards. Participants consented by continuing with participation after reading a study information sheet.

## Author Contributions

CC and BW contributed to study design. CC analyzed all data and drafted the manuscript. BW and RB provided critical revisions.

## Conflict of Interest Statement

The authors declare that the research was conducted in the absence of any commercial or financial relationships that could be construed as a potential conflict of interest.

## Footnotes

1 There are different types of compatibilism/incompatibilism and multiple types of determinism (e.g., Deery et al., 2014), but these are the definitions we are using for the present work.

2 We understand that the word “argument” has a precise meaning in philosophy and can only be evaluated in certain ways (e.g., valid or invalid). In this paper, we use the word “argument” to refer to a statement or reason for or against a point (e.g., Merriam-Webster, Oxford, dictionary.com). We estimated that fewer than 7% of participants in our samples were likely to have taken Critical Thinking courses. Additionally, in open-ended responses, participants did not object or comment on our usage of the word argument or the available evaluations (e.g., “can the argument be true?”).

3 In recent years, sophisticated philosophical thought has developed multiple perspectives on determinism, and so it is increasingly difficult to provide a simple operational definition that laypersons can understand but that all or most philosophers would accept. Our use of the term harks back to Laplace (1814) original thought experiment, which asserted that if a super-intelligent mind knew the disposition of every particle in the universe at any given moment plus all the laws of nature, it could calculate with 100% accuracy the state of the universe at any other point in time. We have emphasized the inevitability aspect as the inspiring but contentious aspect of determinism. Presumably no one thinks that such calculations will be done any time soon, if ever, and indeed the failures of prediction despite accumulated scientific knowledge continue to shock and dishearten social scientists. The possibility of calculating the future with complete accuracy based on knowledge of the present in our view entails that each future event is already inevitable and indeed has been so since time immemorial. Of course, the inevitable future rests on causal processes working through people’s choices and actions, so this differs from the fatalistic view that the future will turn out the same regardless of what oneself does.

4 Here we mean indeterminist in the sense that human actions are not fully determined, not indeterminist in the sense of randomness or uncertainty.

5 For space purposes, exact phrasings of all passages are reported in Supplementary Material.

6 In all studies reporting odds ratios as estimates of effect sizes (Studies 1–3b), classifications of small, medium, and large were interpreted from Chen et al. (2010).

7 Classifications of small, medium, and large effects for studies reporting *r*s or *d*s (mini-meta, Studies 4 and 5) were interpreted using Cohen’s standards (Cohen, 1988).

8 For this outcome, we compared incompatibilists’ reports that the arguments opposed each other to compatibilists’ reports that the arguments supported each other, rather than using the average of the two with one reverse scored (as in the experimental analyses). This was also done for the gut outcome such that we compared incompatibilists’ reports that their gut said the arguments were incompatible to compatibilists’ reports their gut said the arguments were compatible.

9 Preregistration indicated “Whichever comes first (200 participants or the end of the [summer] session).” The end of the summer session came first, resulting in 162 participants.

10 For this and Study 6 (which also used MTurk workers), we included only participants from the United States, with any HIT approval rates, and excluded participants who had participated in any of the lead researcher’s former MTurk studies that had relevance to free will (i.e., there was no overlap between Study 5 and Study 6 participants).

11 If the majority of participants believed in both immaterial souls and scientific determinism, they would be somewhat forced to assert compatibilism, but only 23.8% of the sample were above the midpoint on both soul belief and determinism belief, so this combination of beliefs was relatively uncommon.

12 Classifications of small, medium, and large $\eta_{p}^{2}$ in Study 6 were interpreted from Richardson (2011).
