# Supplementary material for: Forget the Folk: Moral Responsibility Preservation Motives and Other Conditions for Compatibilism
Source: Front Psychol. 2019 Feb 7;10:215. doi: 10.3389/fpsyg.2019.00215 (PMC6374326; doi:10.3389/fpsyg.2019.00215)
Supplement: Supplementary file 2 [file Data_Sheet_2.PDF]

Forget the Folk: Moral Responsibility Preservation Motives and Other Conditions for Compatibilism

Clark, Winegard, & Baumeister, under review

Initiative for Open Science Statement

No participants were excluded from any study. There were no additional undisclosed manipulations or conditions. No analyses were performed before the corresponding data collection was complete. All datasets and syntax will be publicly available. The only variables deleted from the datasets were potential identifiers and some irrelevant demographics (ethnicity in Studies 1 and 2, years speaking English in Studies 31, 3b, 5, and 6, and all open-ended responses). No other data were collected that are not described in the paper. Differences between studies (e.g., samples [mturk adults or undergraduates at various universities], sample size, subject payments) were often due to the location of and resources available for the lead researcher.

Supplemental Materials

Study 1

Methodological Details

Free Will Passage:

> *Take a moment to think about human decision-making. Humans are highly intelligent, complex beings with the capacity for free will. Therefore, humans can be morally responsible for what they do. They sometimes deserve to be praised for good things they freely decide to do and deserve to be blamed for bad things they freely decide to do.*

Determinism Passage:

> *Over the past century, scientists have come to learn the many causes of human behavior, including genes, brain chemistry, and social environment, and have learned that these factors predict human behavior entirely. All human thoughts and their corresponding behaviors are inevitable consequences of prior events. If past events had happened differently, then a person would have had different thoughts and desires, which would have led to different decisions.*

Indeterminism Passage:

> *Over the past century, scientists have come to learn the many causes of human behavior, including genes, brain chemistry, and social environment, but have learned that these factors do not predict human behavior entirely. Human thoughts and their corresponding behaviors are not always inevitable consequences of prior events. Even if past events had happened the exact same way, a person could have different thoughts and desires, which could lead to different decisions.*

Questions (Response options: ‘Yes,’ ‘No,’ and ‘I don’t know’)

1. *According to the argument, do humans’ thoughts influence their behavior?*
2. *According to the argument, if specific events occurred, would a human make a particular decision with 100% certainty*
3. *If humans have free will and can be morally responsible as specified above, is it possible for the argument to be true?*
4. *If humans have free will and can be morally responsible as specified above, is it possible for all human behavior to be inevitable consequences of prior causes?*

Results with Subgroups (all participants, and each group of comprehenders)

Table 1.
Study 1 compatibility response results by condition and subgroups.

Argument Compatibility	*Determinism Condition*	*Indeterminism Condition*	*OR*	*b*	*SE*	*Wald*	*p*	*95% CI*
**All participants**								
Yes	59.3%	82.7%						
No	24.1%	7.7%	4.37	1.47	.62	5.70	.017	1.30, 14.65
Don’t Know	16.7%	9.6%	2.42	.88	.61	2.13	.144	.74, 7.91
**Bypass comprehenders**								
Yes	61.9%	79.1%						
No	23.8%	9.3%	3.27	1.19	.65	3.36	.067	.92, 11.61
Don’t Know	14.3%	11.6%	1.57	.45	.66	.47	.494	.43, 5.71
**Otherwise comprehenders**								
Yes	63.6%	81.0%						
No	13.6%	7.1%	2.43	.89	.88	1.03	.311	.44, 13.52
Don’t Know	22.7%	11.9%	2.43	.89	.71	1.57	.210	.61, 9.72
**Determinism Compatibility**								
**All participants**								
Yes	44.4%	21.2%						
No	25.9%	53.8%	.23	-1.47	.49	9.06	.003	.09, .60
Don’t Know	29.6%	25.0%	.56	-.57	.52	1.21	.272	.20, 1.57
**Bypass comprehenders**								
Yes	50.0%	23.3%						
No	26.2%	51.2%	.24	-1.44	.53	7.25	.007	.08, .68
Don’t Know	23.8%	25.6%	.43	-.84	.58	2.07	.150	.14, 1.35
**Otherwise comprehenders**								
Yes	54.5%	19.0%						
No	18.2%	57.1%	.11	-2.20	.71	9.66	.002	.03, .44
Don’t Know	27.3%	23.8%	.40	-.92	.69	1.77	.184	.10, 1.54

Note. ‘Yes’ response is reference category.

Study 2

Methodological Details

Libertarian Free Will Passage:

> *Over the past century, scientists have come to learn many influences on human behavior, including genes, brain chemistry, and social environment. These are typically beyond human control. However, scientists believe that these uncontrollable factors cannot entirely predict human behavior with 100% certainty, and indeed probably never will. This means that human behaviors are not inevitable consequences of prior causes and that humans have the capacity to choose among alternate courses of action. Though human behavior is often influenced by various uncontrollable causes, ultimately, humans still have options for how to behave.*

Compatibilist Free Will Passage:

> *Over the past century, scientists have come to learn many influences on human behavior, including genes, brain chemistry, and social environment. These are typically beyond human control. Scientists believe these uncontrollable factors entirely predict human behavior with 100% certainty. This means that all human behaviors are inevitable consequences of prior causes and that humans lack the capacity to choose among alternate courses of action. However, humans do have the capacity to act in accordance with their own thoughts and desires. Though these thoughts and desires are entirely determined by uncontrollable causes, they do still contribute to behaviors. Human thoughts and actions are links in a chain of causes.*

Questions (Response options: 'Yes,' 'No,' and 'I don't know')

1a. Neutral Condition: *If these scientists are correct, is it possible for a person to behave differently than that person ultimately does?*
1b. Morally Bad Condition: *If these scientists are correct, is it possible that a man who murdered his wife in order to have an affair with his secretary could have not done so, that is, could he have not murdered his wife?*
1c. Morally Good Condition: *If these scientists are correct, is it possible that a man who donated 90% of his annual salary to a pediatric cancer center could have not done so, that is, could he have not donated 90% of his annual salary to the pediatric cancer center?*

1a. Neutral Condition: *If these scientists are correct, does it make sense to hold people morally responsible for their behavior?*
1b. Morally Bad Condition: *If these scientists are correct, does it make sense to morally blame a man who murdered his wife in order to have an affair with his secretary?*
1c. Morally Good Condition: *If these scientists are correct, does it make sense to morally praise a man who donated 90% of his annual salary to a pediatric cancer center?*

Results with Subgroups (all participants, and comprehenders)

Table 2.
Moral responsibility responses by conditions in Study 2 with subgroups.

		All Participants			Comprehenders		
**Free Will Condition**	**Moral Condition**	**Yes**	**No**	**Don't Know**	**Yes**	**No**	**Don't Know**
Compatibilist Free Will	Good	54.7%	39.1%	6.3%	33.3%	58.3%	8.3%
	Bad	41.7%	50.0%	8.3%	26.1%	73.9%	0.0%
	Neutral	33.9%	46.8%	19.4%	5.3%	89.5%	5.3%
Libertarian Free Will	Good	78.5%	9.2%	12.3%	80.0%	8.0%	12.0%
	Bad	86.4%	8.5%	5.1%	87.5%	10.4%	2.1%
	Neutral	71.2%	13.6%	15.3%	73.6%	13.2%	13.2%

Table 3.
Moral responsibiliity results in Study 2 with subgroups.

	**Comparison**	***OR***	***b***	***SE***	***Wald***	***p***	***95% CI***
**All participants**							
Condition	Yes vs. No	7.95	2.07	.29	50.78	<.001	4.49, 14.06
	Yes vs. DK	1.91	.65	.35	3.46	.063	.97, 3.77
Good dummy	Yes vs. No	1.73	.55	.32	2.88	.090	.92, 3.24
	Yes vs. DK	2.44	.89	.40	4.94	.026	1.11, 5.34
Bad dummy	Yes vs. No	1.32	.27	.32	.73	.392	.70, 2.46
	Yes vs. DK	3.20	1.16	.45	6.64	.010	1.32, 7.75
**Bypass comprehenders**							
Condition	Yes vs. No	28.83	3.36	.42	62.77	<.001	12.55, 66.20
	Yes vs. DK	1.86	.62	.71	.75	.386	.46, 7.51
Good dummy	Yes vs. No	3.01	1.10	.51	4.75	.029	1.12, 8.12
	Yes vs. DK	1.28	.25	.55	.21	.651	.44, 3.79
Bad dummy	Yes vs. No	2.02	.70	.49	2.07	.150	.78, 5.25
	Yes vs. DK	10.01	2.30	1.08	4.51	.034	1.20, 83.84

Note. 'DK' stands for 'Don't Know' response.

Study 3a

Methodological Details

Determinism argument:

Over the past century, scientists have come to learn the many causes of human behavior, including genes, brain chemistry, and social environment, and have learned that these factors predict human behavior entirely. All human thoughts and their corresponding behaviors are inevitable consequences of prior events. People's mental states (their beliefs, desires, and decisions) do have an effect on what they do, but those mental states are merely part of the causal chains that lead to their actions. While people can behave according to their thoughts and desires, no human is ever capable of having different thoughts or behaving in any way different than they ultimately do. From before a person is even born, every single thought they will ever have and every single action they will ever perform has already been predetermined.

Morally Neutral Condition: *So, for example, if a man decides to have pasta for dinner and subsequently has pasta for dinner, given his genes, brain chemistry, and his social history, there is no way he could have not had pasta for dinner.*

Morally Bad Condition: *So, for example, if a man decides to rob a convenience store and subsequently robs a convenience store, given his genes, brain chemistry, and his social history, there is no way he could have not robbed the convenience store.*

Free will argument:

We must appreciate the full richness and complexity of human decision-making. While many external and internal factors interact to influence human behavior, humans are capable of free action. Because humans have free will, they deserve to be held responsible for their behavior. When a person behaves in a particular way, they are responsible for having behaved that way.

Morally Neutral Condition: *So, for example, if a man has pasta for dinner, he exercised his free will in having pasta for dinner and ought to be considered responsible for that action.*

Morally Bad Condition: *So, for example, if a man robs a convenience store, he exercised his free will in robbing a convenience store and ought to be held morally responsible for that action.*

Questions

1. For this question, we are not interested in your agreement with each argument or whether you personally believe either argument is true. We simply want to know whether you think the second argument is consistent or inconsistent with the first argument. That is, if the first argument is true (*humans have free will and can be held morally responsible/humans are incapable of behaving in any way other than they ultimately do)*, does that information

automatically mean the second argument is untrue (*humans have free will and can be held morally responsible/humans are incapable of behaving in any way other than they ultimately do)*? In other words, are the arguments inconsistent, or is it possible both arguments could be correct (they are consistent)? Again, do not report whether you think the second argument is true, but whether it can possibly be true if the first argument is true.

Response options:

'If the first argument is true (that all human thoughts and actions are predetermined/that humans have free will and are morally responsible for their behavior), the second argument (that humans have free will and are morally responsible for their behavior/that all human thoughts and actions are predetermined) CAN also be true'

'If the first argument is true (that all human thoughts and actions are predetermined/that humans have free will and are morally responsible for their behavior), the second argument (that humans have free will and are morally responsible for their behavior/that all human thoughts and actions are predetermined) CANNOT also be true'

'I don't know'

2. How certain are you that your answer is correct? (Response options: 'Not at all' to 'Entirely' on a 100-point sliding scale)
3. According to (the determinism) argument, do people's desires, beliefs, and decisions have an effect on what they do? (Response options: 'Yes,' 'No,' and 'I don't know')
4. According to (the determinism) argument, is it possible for a human to behave in any way other than they ultimately do? (Response options: 'Yes,' 'No,' and 'I don't know')

Results with Subgroups (all participants, and each group of comprehenders)

Table 4.
Compatibility responses by condition in Study 3a with subgroups.

		All Participants			Bypass Comprehenders			Otherwise Comprehenders		
**Order Condition**	**Moral Condition**	**Yes**	**No**	**DK**	**Yes**	**No**	**DK**	**Yes**	**No**	**DK**
Free Will First	Moral	41.6%	56.2%	2.2%	49.2%	50.8%	0.0%	37.5%	62.5%	0.0%
	Not Moral	33.7%	65.1%	1.2%	37.1%	61.3%	1.6%	22.8%	77.2%	0.0%
Free Will Second	Moral	20.7%	72.4%	6.9%	22.0%	72.9%	5.1%	13.6%	81.8%	4.5%
	Not Moral	33.3%	61.9%	4.8%	38.8%	58.2%	2.0%	30.2%	66.0%	3.8%

Note. 'DK' stands for 'Don't know' response.

Table 5.
Compatibility results in Study 3a with subgroups.

	Comparison	*OR*	*b*	*SE*	*Wald*	*p*	*95% CI*
**All participants**							
Order Condition	Yes vs. No	.96	-.04	.33	.01	.905	.51, 1.83
	Yes vs. DK	4.14	1.42	1.15	1.53	.216	.44, 39.39
Moral Condition	Yes vs. No	.53	-.63	.36	3.18	.075	.26, 1.07
	Yes vs. DK	.43	-.85	.71	1.41	.234	.11, 1.73
Interaction	Yes vs. No	2.69	.99	.48	4.35	.037	1.06, 6.83
	Yes vs. DK	1.49	.40	1.44	.08	.782	.09, 24.96
**Bypass comprehenders**							
Order Condition	Yes vs. No	.92	-.08	.40	.04	.841	.43, 2.01
	Yes vs. DK	1.21	.19	1.45	.02	.895	.07, 20.67
Moral Condition	Yes vs. No	.46	-.77	.43	3.19	.074	.20, 1.08
	Yes vs. DK	1.21	.19	1.45	.02	.895	.07, 20.67
Interaction	Yes vs. No	3.47	1.25	.56	4.86	.027	1.15, 10.50
	Yes vs. DK		**cannot be estimated due to small cells**				
**Oherwise comprehenders**							
Order Condition	Yes vs. No	.65	-.44	.44	1.00	.318	.28, 1.52
	Yes vs. DK		**cannot be estimated due to small cells**				
Moral Condition	Yes vs. No	.37	-1.01	.47	4.61	.032	.15, .92
	Yes vs. DK	.38	-.98	1.00	.96	.328	.05, 2.68
Interaction	Yes vs. No	5.57	1.72	.63	7.44	.006	1.61, 19.14
	Yes vs. DK		**cannot be estimated due to small cells**				

Note. 'DK' stands for 'Don't Know' response. 'Yes' response is the reference condition.

All participants:

"In the morally neutral condition, participants were no more likely to be compatibilist when evaluating whether free will is compatible with determinism than when evaluating whether determinism is compatible with free will (34.1% vs. 35%), $\chi^2 = .01$, $p = .905$. In the morally relevant condition, participants were more compatibilist when evaluating whether determinism can be true if people have free will than when evaluating whether people can have free will if determinism is true (42.5% vs. 22.2%), $\chi^2 = 7.86$, $p = .005$."

Bypass comprehenders:

Neutral condition: (Free Will First 37.7% vs. Free Will Second 39.6%), $\chi^2 = .04$, $p = .841$
Moral condition: (Free Will First 49.2% vs. Free Will Second 23.2%), $\chi^2 = 8.72$, $p = .003$

Otherwise comprehenders:

Neutral condition: (Free Will First 22.8% vs. Free Will Second 31.4%), $\chi^2 = 1.01$, $p =$ .316
Moral condition: (Free Will First 37.5% vs. Free Will Second 14.3%), $\chi^2 = 8.47$, $p = .004$

Study 3b

Methodological Details

Identical to Study 3a, but morally bad condition only, no certainty question, and the addition of these questions:

We are interested in how you came to your conclusion on the previous page. Likely, you had many different thoughts and considerations that influenced your decision. Below is a list of various thoughts people have when considering such arguments. Please indicate the extent to which the thought influenced your conclusion. (Response options: 'Did not influence my conclusion at all' to 'Completely influenced my conclusion' on 9-point scales)

1. Human thoughts and actions ARE predetermined
2. Human thoughts and actions ARE NOT predetermined
3. Humans DO have free will
4. Humans DO NOT have free will
5. Humans ARE responsible for their behavior
6. Humans ARE NOT responsible for their behavior
7. My gut says these arguments ARE compatible
8. My gut says these arguments ARE NOT compatible
9. These arguments directly support each other
10. These arguments directly oppose each other
11. These arguments are unrelated to each other
12. I do not understand these arguments

Results with Subgroups (all participants, and each group of comprehenders)

Table 6.
Compatibility responses by condition in Study 3b with subgroups.

	**All Participants**			**Bypass Comprehenders**			**Otherwise Comprehenders**		
**Order Condition**	**Yes**	**No**	**DK**	**Yes**	**No**	**DK**	**Yes**	**No**	**DK**
Free Will First	26.6%	69.1%	4.3%	32.8%	64.1%	3.1%	24.3%	74.3%	1.4%
Free Will Second	21.4%	77.6%	1.0%	32.8%	67.2%	0.0%	18.2%	81.8%	0.0%

Note. 'DK' stands for 'Don't know' response.

Table 7.
Compatibility results in Study 3b with subgroups.

**All participants**	***OR***	***b***	***SE***	***Wald***	***p***	***95% CI***
Yes vs. No	1.39	.33	.34	.94	.332	.71, 2.72
Yes vs. DK	.30	-1.21	1.16	1.10	.295	.03, 2.87
**Bypass comprehenders**						
Yes vs. No	1.05	.05	.39	.02	.897	.49, 2.25
Yes vs. DK		**cannot be estimated due to small cells**				
**Oherwise comprehenders**			.43	3.19	.074	.20, 1.08
Yes vs. No	1.47	.39	.41	.90	.342	.66, 3.26
Yes vs. DK		**cannot be estimated due to small cells**				

Note. 'DK' stands for 'Don't Know' response. 'Yes' response is the reference condition.

Study 4

Methodological Details

Determinism Passage:

Everything that happens in the universe is completely caused by what happened before it. This has been true from the very beginning of the universe, so what happened in the beginning of the universe caused what happened next, and so on right up until the present. If the universe was re-created over and over again, starting from the exact same initial conditions with all the same laws of nature, this would cause the exact same events for the entire history of the universe, so that every single time the universe was re-created, everything would happen the exact same way, including human decision-making.

This does not mean that people's mental states have no effect on what they end up doing. Rather, people's mental states are part of the causal chains that lead to their actions, though their mental states are always completely caused by earlier things in the causal chain that happened before them. Given the initial conditions of the universe and the laws of nature, whenever a person decides to do something, it had to happen that that person would decide to do the exact same thing at the exact same time and then do it.

For example, one day John decided to rob a convenience store. Like everything else in the universe, his decision was completely caused by what happened before it. Given the initial conditions of the universe and the laws of nature, it had to happen that John would decide to rob that convenience store.

Free Will is Unnecessary for Moral Responsibility Passage:

A great deal of time and attention has been put forth to determine whether free will does or does not exist. However, recently, philosophers and scientists are realizing that free will is not a requirement for holding people morally responsible. As can be observed in the most basic psychological studies, even rats' and pigeons' behavior can be altered with reinforcement and punishment. These same concepts can be applied to humans. Whether or not humans have free will, we can still hold people morally responsible

purely for purposes of deterring bad behavior. In fact, in a universe that is completely determined by prior causes, punishment may be considered one of the environmental factors contributing to human behavior. If humans know that bad behavior will be punished, they will be less likely to perform that bad behavior. Holding people morally responsible is not an issue of free will; it is merely a means of discouraging behavior destructive to society.

Questions

1. If the argument is correct, can people have free will? (Response option: 'Definitely no' to 'Definitely yes' on 7-point scale)
2. If the argument is correct, can people be held morally responsible? (Response option: 'Definitely no' to 'Definitely yes' on 7-point scale)
3. Does the argument support or oppose the existence of human free will? (Response options: 'Supports,' 'Opposes,' 'Neither supports or opposes')
4. Does the argument support or oppose the existence of human moral responsibility? (Response options: 'Supports,' 'Opposes,' 'Neither supports or opposes')
5. According to this argument, do people's beliefs and desires have an effect on what they do? Response options: 'Yes,' 'No,' and 'I don't know')
6. According to this argument, given the laws of nature and the initial conditions of the universe, do later events (including human decisions) have to happen as they do? Response options: 'Yes,' 'No,' and 'I don't know')

Results with Subgroups (all participants, and each group of comprehenders)

All participants:

"There was a very small effect such that participants reported that free will was less compatible with determinism after reading that free will is unnecessary for moral responsibility (M= 3.97, SD = 2.11) than before (M= 3.76, SD = 2.02), t(161) = 1.66, p = .098, Cohen's d = .131, while there was virtually no effect on moral responsibility judgments (Time 1 M= 4.41, SD = 2.12, Time 2 M= 4.33, SD = 1.94), t(161) = .57, p = .570, Cohen's d = .049."

Bypass comprehenders:

Free Will Time 1: M= 4.48, SD = 1.94
Free Will Time 2: M= 4.28, SD = 1.93, t(93) = 1.28, p = .203, Cohen's d = .131
Moral Responsibility Time 1: M= 5.06, SD = 1.87
Moral Responsibility Time 2: M= 4.89, SD = 1.84, t(93) = 1.11, p = .270, Cohen's d = .115

Otherwise comprehenders:

Free Will Time 1: M= 3.66, SD = 2.06
Free Will Time 2: M= 3.49, SD = 2.00, t(123) = 1.13, p = .260, Cohen's d = .102
Moral Responsibility Time 1: M= 4.27, SD = 2.10
Moral Responsibility Time 2: M= 4.19, SD = 1.98, t(123) = .50, p = .615, Cohen's d = .050

Participants who comprehended that the argument that opposed the necessity of free will for moral responsibility neither opposed or supported the existence of human free will:

Free Will Time 1: *M*= 3.54, *SD* = 2.06

Free Will Time 2: *M*= 3.66, *SD* = 2.10, *t*(84) = -.71, *p* = .483, Cohen's *d* = -.078

Moral Responsibility Time 1: *M*= 4.20, *SD* = 2.19

Moral Responsibility Time 2: *M*= 4.15, *SD* = 2.01, *t*(84) = .27, *p* = .787, Cohen's *d* = .031

Study 5

Methodological Details

Determinism argument:

Science has come to learn that we live in a deterministic universe. This means that everything that happens is completely caused by whatever happened before it. This is true from the very beginning of the universe. What happened in the beginning of the universe caused what happened next, and so on right up until the present. People's mental states (their beliefs, desires, and decisions) do have an effect on what people end up doing, but these mental states are part of the causal chains that lead to their actions. Mental states are always completely caused by earlier things in the causal chain that happened before them. Given that the past happened the way it did, all human decisions ever made had to happen the way they did.

Soul argument:

The human experience is very complex. While many external and internal factors interact to influence human behavior, human experience cannot be reduced to purely physical causes. Even if we understood all physical causes, we could not fully understand or predict human thoughts or behaviors. This suggests that humans have an immaterial soul, which is different from the physical world. The human individual is part physical body, part immaterial soul. When people die, the body continues to physically exist, but your soul is no longer present in the body. This soul is the source of each individual's unique, subjective experience of the world, and his or her identity. It is what makes each of us who we are. Death is not the end but rather a separation of the soul from the physical body.

Questions

1. If we live in a completely deterministic universe, is it possible that humans have immaterial souls? (Response options: 'Definitely no' to 'Definitely yes' on a 100-point sliding scale)
2. Do you personally agree with this argument? That is, do you think that we live in a completely deterministic universe? (Response options: 'Yes,' 'No,' and 'I don't know')
3. Do you personally agree with this argument? That is, do you think that humans have immaterial souls? (Response options: 'Yes,' 'No,' and 'I don't know')

4. According to (the determinism) argument, do people's desires, beliefs, and decisions have an effect on what they do? (Response options: 'Yes,' 'No,' and 'I don't know')
5. According to (the determinism) argument, is it possible for a human to behave in any way other than they ultimately do? (Response options: 'Yes,' 'No,' and 'I don't know')

Results with Subgroups (all participants, and each group of comprehenders)

All participants:

"People were generally soul compatibilist (M = 60.64, SD = 30.34, 65.3% of participants above the midpoint), which was statistically different from zero, $t(100) = 20.09$, $p < .001$, and significantly above the midpoint, $t(100) = 3.36$, $p = .001$."

Bypass comprehenders:

M = 60.45, SD = 30.05, 65.1% of participants above the midpoint, which was statistically different from zero, $t(85) = 18.66$, $p < .001$, and significantly above the midpoint, $t(85) = 3.07$, $p = .003$.

Otherwise comprehenders:

M = 58.80, SD = 31.56, 61.3% of participants above the midpoint, which was statistically different from zero, $t(75) = 16.13$, $p <.001$, and significantly above the midpoint, $t(74) = 2.28$, $p = .026$.

All participants:

"Stronger belief in souls ($r = .37$, $p < .001$) and stronger belief in determinism ($r = .26$, $p = .009$) were also associated with stronger soul compatibilism."

Bypass comprehenders:

Belief in souls: $r = .50$, $p < .001$
Belief in determinism: $r = .25$, $p =.020$

Otherwise comprehenders:

Belief in souls: $r = .27$, $p = .022$
Belief in determinism: $r = .22$, $p =.054$

Study 6

Methodological Details

Primate free will argument:

When it comes to decision-making, human beings and other primates (e.g., other great apes, monkeys) have a great deal in common. Studies suggest that chimpanzees do show some degree of self-awareness and can anticipate how their actions will impact the environment around them. While chimpanzees and other great apes do show some capacity to reason, their ability to understand cause-and-effect relationships approximates that of human toddlers. This all raises the question of whether chimpanzees

and other primates have "free will". In considering the similarities and differences in cognitive functioning between humans and other primates, we must conclude that if other primates have free will, then the free will that makes people personally responsible for their behavior does (not really/indeed) exist.

Questions (Response options: 'Definitely no' to 'Definitely yes' on a 100-point sliding scale)

1. Do you believe that **other primates** have free will?
2. Do the arguments support the conclusion that ***other primates*** *have free will* or *do not have free will*?
3. Do you believe **humans** have free will?
4. Do the arguments support the conclusion that ***humans*** *have free will* or *do not have free will*?
